# Heterologous expression of 2-methylisoborneol / 2 methylenebornane biosynthesis genes in *Escherichia coli* yields novel C11-terpenes

**DOI:** 10.1371/journal.pone.0196082

**Published:** 2018-04-19

**Authors:** Max J. Kschowak, Hannah Wortmann, Jeroen S. Dickschat, Jens Schrader, Markus Buchhaupt

**Affiliations:** 1 DECHEMA Research Institute, Industrial Biotechnology, Frankfurt am Main, Germany; 2 Faculty of Biological Sciences, Goethe University, Frankfurt am Main, Germany; 3 Kekulé-Institute of Organic Chemistry and Biochemistry, Rheinische Friedrich Wilhelms University, Bonn, Germany; Michigan State University, UNITED STATES

## Abstract

The structural diversity of terpenoids is limited by the isoprene rule which states that all primary terpene synthase products derive from methyl-branched building blocks with five carbon atoms. With this study we discover a broad spectrum of novel terpenoids with eleven carbon atoms as byproducts of bacterial 2-methylisoborneol or 2-methylenebornane synthases. Both enzymes use 2-methyl-GPP as substrate, which is synthesized from GPP by the action of a methyltransferase. We used *E*. *coli* strains that heterologously produce different C11-terpene synthases together with the GPP methyltransferase and the mevalonate pathway enzymes. With this *de novo* approach, 35 different C11-terpenes could be produced. In addition to eleven known compounds, it was possible to detect 24 novel C11-terpenes which have not yet been described as terpene synthase products. Four of them, 3,4-dimethylcumene, 2-methylborneol and the two diastereomers of 2-methylcitronellol could be identified. Furthermore, we showed that an *E*. *coli* strain expressing the GPP-methyltransferase can produce the C16-terpene 6-methylfarnesol which indicates the condensation of 2-methyl-GPP and IPP to 6-methyl-FPP by the *E*. *coli* FPP-synthase. Our study demonstrates the broad range of unusual terpenes accessible by expression of GPP-methyltransferases and C11-terpene synthases in *E*. *coli* and provides an extended mechanism for C11-terpene synthases.

## Introduction

Terpenoids constitute the largest class of natural products with more than 70,000 compounds that have been discovered so far [1]. The diversity of structures is reflected in its various applications as nutrients (carotenoids), drugs (artemisinin and taxol), potential biofuels (farnesane), and flavor and fragrance compounds ((-)-menthol, sclareol) (for a comprehensive overview see [2], in particular part 3).

However, the structural diversity is limited by the isoprene rule, which states that terpenes have a methyl-branched carbon chain with a length of a multiple of five carbons. Already in 1887 Otto Wallach concluded during his comprehensive work on structures of terpenoids that terpenes are formal oligomers of isoprene [3]. In 1953 Leopold Ružička extended the isoprene rule and described non-canonical terpenoids as the products of subsequent reactions on canonical terpenes [4]. The cellular C5 precursors of all terpenes are isopentenyl diphosphate (IPP) and its isomer dimethylallyl diphosphate (DMAPP). They are produced by two independently evolved pathways: the mevalonate pathway and the later discovered 2-C-methyl-d-erythritol 4-phosphate pathway. They proceed *via* different intermediates, but culminate in the same end products.

**Fig 1:**
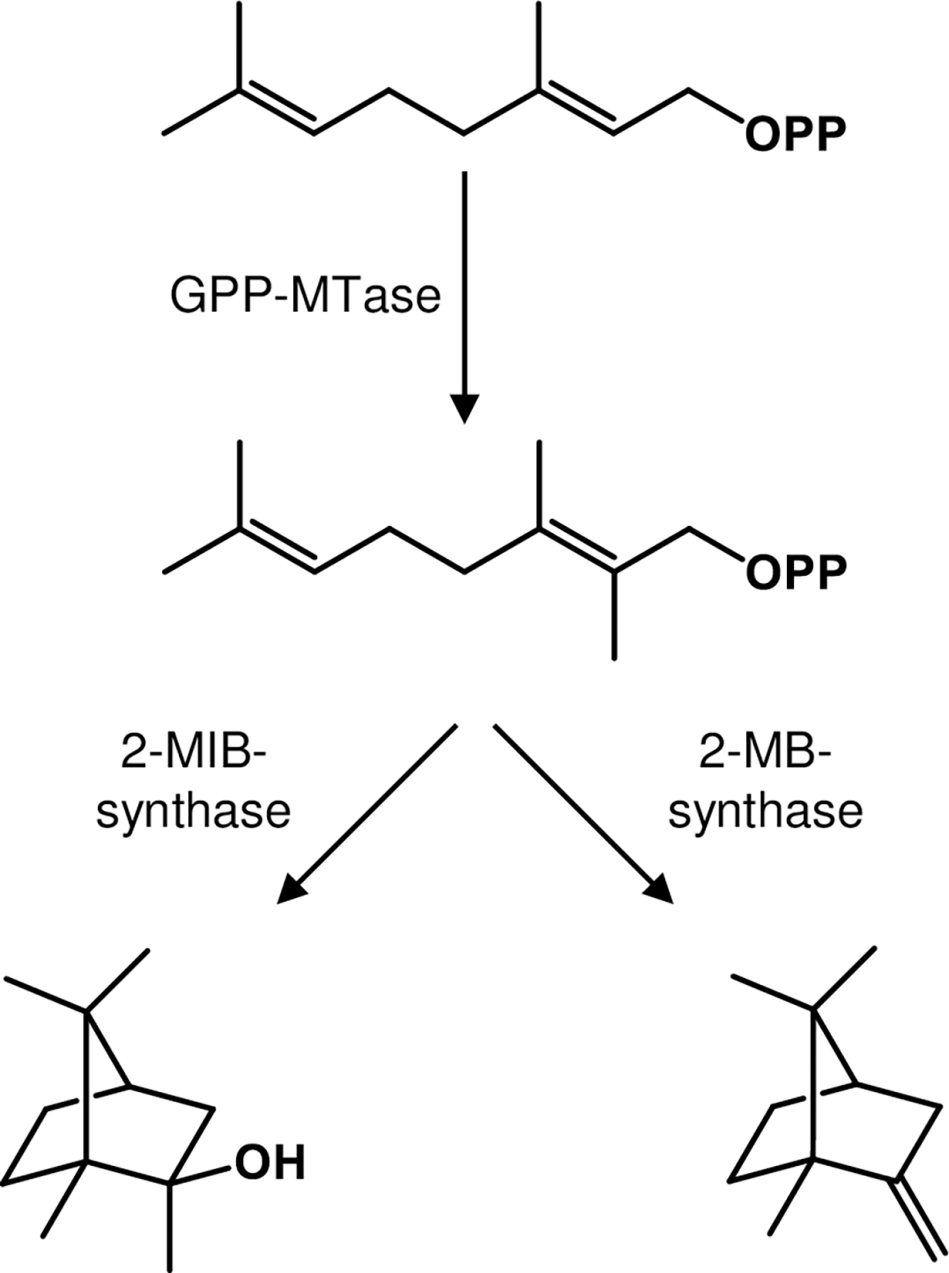
Biosynthesis of 2-methylisoborneol (2-MIB) and 2-methylenebornane (2-MB) GPP is methylated at the second C-atom by a GPP-MTase. 2-methyl-GPP serves as substrate for the 2-MIB synthase and the 2-MB synthase.

In 2007 and 2008 the elucidation of the biosynthetic pathway to the non-canonical terpene and off-flavor 2-methylisoborneol (2-MIB) in different microorganisms was reported independently by three research groups, based on feeding experiments with isotopically labelled precursors [5] or on *in vitro* experiments with recombinant enzymes [6,7]. In a first step the S-adenosyl-l-methionine-dependent geranyl diphosphate C-methyltransferase (GPP-MTase) catalyzes the electrophilic methylation at the C-2 position of geranyl diphosphate (GPP). The 2-methyl-GPP serves as substrate for the 2-MIB synthase, which catalyzes the cyclization to the bicyclic terpene alcohol 2-MIB (Fig 1). The crystal structures of both involved enzymes have been determined and catalytic mechanisms of the GPP-MTase and 2-MIB synthase were proposed [8,9].

2-MIB has a muddy smell with a low odor and flavor threshold (< 5 ng/L) [10,11] and is a contaminant of drinking water, wine [12] and fish [13]. Moreover it is related to the musty-earthy aroma in camembert and brie [14]. Since its isolation from streptomycetes and structure elucidation in 1969 [15,16], many other bacteria were identified as producers. Mainly actinomycetes and cyanobacteria [17], but also myxobacteria [5] and eukaryotes like fungi [14,18] and liverwort [19] were reported to release 2-MIB.

Besides 2-MIB synthases, two terpene synthases (TS) are described that produce the C11-terpene 2-methylenebornane (2-MB) as main product. Likewise, the 2-MB synthases of *Pseudomonas fluorescens* and *Micromonospora olivasterospora* use 2-methyl-GPP as substrate and are closely related to 2-MIB synthases [6,20].

The reaction from 2-methyl-GPP to 2-MIB or to 2-MB proceeds via a series of carbocationic intermediates. Corresponding to other terpene synthases these carbocations can be quenched by the addition of water or by deprotonation [21]. Early quenching reactions at the cationic intermediates of the 2-MIB cyclization cascade explain the previously identified side products of C11-terpene synthases (C11-TS) from different bacteria, including 2-methylgeraniol, 2-methylnerol, 2-methyllinalool, 2-methylmyrcene, 2-methyl-α-terpineol, 2-methyl-β-fenchol, 2-methyllimonene, 2-methyl-2-bornene and 1-methylcamphene [20,22,23]. The 2-methylbornyl cation is the last carbocation in the reaction mechanism catalyzed by C11-TSs and is the precursor for the two different main products 2-MIB or 2-MB.

In this study we heterologously expressed four different C11-TS genes, in each case together with the genes for a GPP-MTase and for the whole mevalonate pathway including an IPP-isomerase gene, in *E*. *coli* to produce C11-terpenes. Using headspace-solid phase micro extraction (HS-SPME) or stir bar sorptive extraction (SBSE) and subsequent gas chromatography-mass spectrometry (GC-MS) analysis we observed the production of many different compounds and were able to identify diverse mono-, C11-, sesqui- and C16-terpenoids from the *E*. *coli* strains containing such a non-canonical terpenoid biosynthesis pathway.

## Materials and methods

*E*. *coli* strain DH5α (New England Biolabs) was used for plasmid construction. *E*. *coli* MG1655 Δ*endA* Δ*recA* (DE3) (hereinafter referred to as *E*. *coli* MG1655) [24] was constructed in the lab of Kristala Prather (Addgene #37854) and used for production experiments. pJBEI-6409 was constructed in the lab of Taek Soon Lee (Addgene plasmid # 47048). It contains the genes of the mevalonate pathway, of an IPP isomerase, of a GPP synthase and of a limonene synthase [25] and was used to construct a plasmid for provision of high GPP levels.

*E*. *coli* strains were cultivated routinely at 37°C in lysogeny broth (LB, 10 g/L yeast extract, 10 g/L NaCl, 5 g/L tryptone [26]) containing the appropriate antibiotics (ampicillin, 100 μg/mL; chloramphenicol, 34 μg/mL; gentamicin, 20 μg/mL).

Genes encoding the 2-MB synthase from *Pseudomonas fluorescens* Pf0-1 (MBSp), the 2-MB synthase from *Micromonospora olivasterospora* (MBSm), the 2-MIB synthase from *Streptomyces griseus subsp*. *griseus* (MIBSg), the 2-MIB synthase from *Streptomyces coelicolor* A3(2) (MIBSc) and the GPP-MTase from *Streptomyces coelicolor* A3(2) were codon-optimized for *E*. *coli* and synthesized by Life Technology GmbH (Darmstadt, Germany). Optimal ribosomal binding sites (RBS, S1 Table) for those genes were designed with the RBS calculator V1.1 [27,28]. New RBSs of C11-TSs were synthesized together with the genes. The new RBS of the GPP-MTase was integrated via Gibson assembly.

2-Methylisoborneol and 2-Methylborneol were synthesized from camphor as previously described [16]. Other C11- and C16-terpene reference substances were synthesized by Enamine Ltd (Riga, Latvia).

### Plasmid construction

The gene for chloramphenicol resistance in pJBEI-6409 were exchanged with the gene for gentamicin resistance via Gibson assembly [29]. The backbone was amplified with the primers mk20 and mk23 (S1 Table), the insert was amplified with the primer pair mk24 / mk25 and pBBR1MCS-5 [30] as template. From the new plasmid pMK-04 (Table 1) the gene encoding the limonene synthase was deleted via Gibson assembly for construction of pMK-05. The backbone was amplified with the primers mk7 and mk8. The oligo mk78x was used as insert. To provide the empty vector control pMK-06, a PCR with the primers mk8 and mk26, a restriction digest of the PCR product with *Bam*HI and self-ligation was done.

**Table 1:** Plasmids used in this study.

Name	Description (origin of replication, antibiotic marker, promoter and genes)	Expressed proteins	Reference
pETDuet-1	colE1, Amp^r^, P_T7_	-	Novagen
pJBEI-6409	p15A, Cm^r^, P_lacUV5_, P_trc_ *atoB*, *mvaS*, *mvaA*, *ERG12*, *ERG8*, *MVD1*, *idi*, *trGPPS2*, *trLS*	Mevalonate pathway proteins, IPP-isomerase, GPP-synthase, limonene synthase	[25]
pMK-04	pJBEI-6409 with Gm^r^ instead of Cam^r^	Mevalonate pathway proteins, IPP-isomerase, GPP-synthase, limonene synthase	This study
pMK-05	pMK-04, *trLS* deleted	Mevalonate pathway proteins, IPP-isomerase, GPP-synthase	This study
pMK-06	pMK-04, empty vector	-	This study
pMK-03	pETDuet-1, *gppmtase*^a^	GPP-MTase (NP_631739.1)	This study
pMK-08	pETDuet-1, *mbsp*^a^	MBSp (WP_011333305.1)	This study
pMK-12	pETDuet-1, *mbsp*, *gppmtase*	MBSp, GPP-MTase	This study
pMK-13	pETDuet-1, *mbsm*^a^, *gppmtase*	MBSm (BAK26793.1), GPP-MTase	This study
pMK-14	pETDuet-1, *mibsg*^a^, *gppmtase*	MIBSg (WP_012378420.1), GPP-MTase	This study
pMK-15	pETDuet-1, *mibsc*^a^, *gppmtase*	MIBSc (NP_733742.1), GPP-MTase	This study

^a^ Optimized gene sequences can be found in S1–S5 Figs

For integration of the GPP-MTase gene into the second multiple cloning site of pETDuet-1, it was amplified with the primers mk12 and mk13. The backbone was amplified with the primers mk10 and mk11. The products were combined to construct plasmid pMK-03 via Gibson assembly.

The genes encoding MBSp, MBSm, MIBSg and MIBSc were integrated in the first multiple cloning sites of pMK-09 and pETDuet-1 via standard restriction cloning (*Xba*I and *Not*I) to provide the plasmids pMK-08, -12, -13, -14 and -15.

### Strains

To construct the production strains (11-p, 11-m, 11-g, 11-c; Table 2) which heterologously express the genes of the mevalonate pathway, of an IPP isomerase, of a GPP synthase, of the GPP-MTase and one of four different C11-TS genes, the respective plasmids were transformed into *E*. *coli* MG1655. Furthermore, control strains lacking a C11-TS gene (11–0), the C11-TS gene and the GPP-MTase gene (10–0) and a negative control with empty vectors (0–0) were constructed.

**Table 2:** Strains used in this study.

Strain	Genotype / harboring plasmids	Reference
*E*. *coli* MG1655	F^-^, λ^-^, *ilvG- rfb-50 rph-1*, Δ*endA*, Δr*ecA*	[24]
*E*. *coli* DH5α	F^-^, Φ80*lacZ*ΔM15, Δ(*lacZYA-argF*)U169, *deoR*, *recA1*, *endA1*,*hsdR17*(r^-^, m^+^), *phoA*, *sup*E44, λ^-^, *thi-1*	[31]
0–0	*E*. *coli* MG1655 + pMK-04 + pETDuet-1	This study
10–0	*E*. *coli* MG1655 + pMK-05 + pETDuet-1	This study
11–0	*E*. *coli* MG1655 + pMK-05 + pMK-03	This study
11-p	*E*. *coli* MG1655 + pMK-05 + pMK-12	This study
11-m	*E*. *coli* MG1655 + pMK-05 + pMK-13	This study
11-g	*E*. *coli* MG1655 + pMK-05 + pMK-14	This study
11-c	*E*. *coli* MG1655 + pMK-05 + pMK-15	This study

### *De novo* terpenoid production in *E*. *coli*

Pre-cultures in reaction tubes containing 5 mL LB medium with appropriate antibiotics were incubated overnight at 37°C and 180 rpm. Main cultures in 15 ml 2x YT medium (16 g/L tryptone, 10 g/L yeast extract, 5 g/L NaCl, pH 7,0 [32]) with 2% (v/v) glycerol and appropriate antibiotics in 100 ml baffled shake flasks were inoculated from pre-cultures to an OD_600_ value of 0.1. After cultivation at 37°C to an OD_600_ value of 1, gene expression was induced with isopropyl-β-D-1-thiogalactopyranoside (IPTG, 100 μM). Induced cultures had terpenoids extracted after 24 hours of incubation at 30°C and 180 rpm.

### HS-SPME GC-MS analysis

Volatile compounds in the headspace of each culture were analyzed by extraction with an 85 μm SPME stableflex fiber composed of polydimethylsiloxane and Carboxen (Supelco, Bellefonte, USA). The SPME fibers were exposed for 30 minutes and then inserted into the injection port of a GC-MS-QP2010 (Shimadzu) containing a DB-5 column (30 m x 0.25 mm x 0.25 μm, Agilent, Santa Clara, USA). Measurements were conducted as follows: helium as carrier gas, splitless injections at 250°C, 1 minute sampling time and column flow of 1.1 mL/min. The column temperature was programmed as follows: 40°C for 1.5 minutes, 10°C/min up to 250°C followed by 20°C/min up to 300°C.

Compounds were identified via comparison of mass spectra and retention indexes (RI) to the ones of reference substances or mass spectra of the NIST mass spectral library (v14) and RIs published by Adams [33]. Identity was assumed if the similarity index was equal to or higher than 90 and the RI was ± 10 compared to the published data.

To identify 2-methyl-α-terpineol ion trace analyses were done. For this purpose, the proportions of the peak areas of the fragment ions at *m/z* = 107, 135, 150 and 93 were compared to the proportions of the signal intensities of the same fragment ions of the reference compound.

Structural conversions of analytes during the procedure were excluded by HS-SPME GC-MS analysis with the mono- and C11-terpenoids.

### SBSE GC-MS analysis

For extraction of less volatile terpenoids, a twister (10 x 0.5 mm PDMS; Gerstel, Mühlheim an der Ruhr, Germany) was fixated in a shake flask by an external magnet in order to cover it completely with culture. After the production phase the twister was removed from the culture, rinsed with ddH_2_O, dried with lint free wipes and transferred to the inlet of a GC-vial, which was filled with 250 μL n-pentane. After 15 minutes of incubation in an ultrasonic bath (Merck eurolab, Darmstadt, Germany) 5 μL of the pentane were used for GC-MS analysis. Besides the injection mode (split ratio = 10) the same settings and equipment described for the HS-SPME GC-MS analysis were used.

## Results

### C11-terpenoids

Volatiles of four different production strains (11-p, 11-m, 11-g and 11-c) heterologously expressing the genes of the mevalonate pathway, an IPP isomerase gene, a GPP synthase gene, the GPP-MTase gene and one of four different C11-TS genes were analyzed with HS-SPME GC-MS.

**Fig 2:**
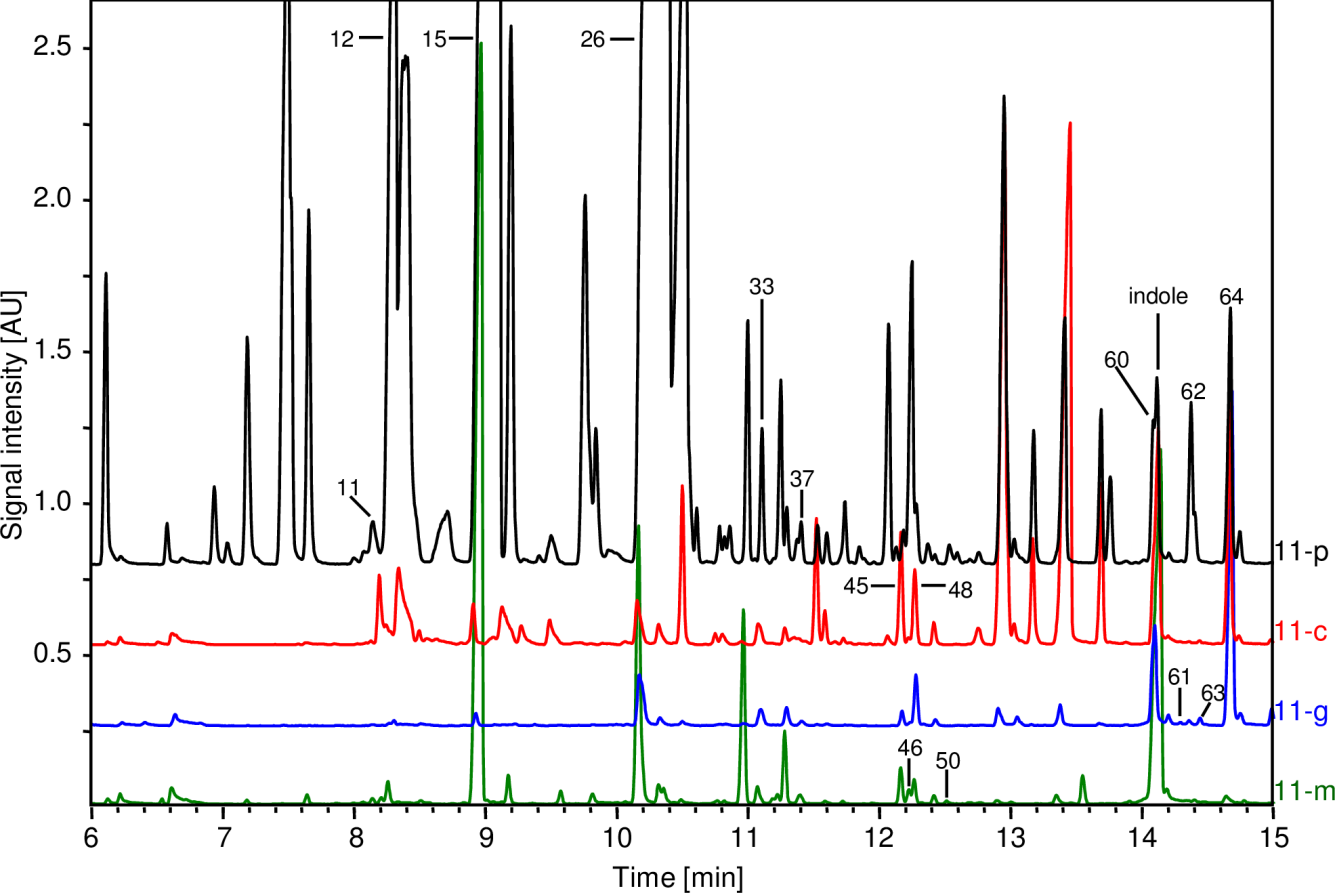
Total ion chromatograms of HS-SPME-GCMS analyses of four production strains expressing four different C11-TSs. The peaks of all identified and mentioned compounds are labeled. Compound names are listed in Table 3.

The resulting total ion chromatograms (Fig 2) and the corresponding mass spectra (S6 Fig) show a complex and diverse mixture of volatiles in the culture headspaces.

From mass spectral data, 35 C11-terpenes could be identified (Table 3). They show typical fragment ions for C11-terpenes e.g. at *m*/*z* 69, 93, 107, 121 and 135 (S6 Fig). Ten of them have a molecular ion of a C11-terpene alcohol at *m*/*z* 168 as observed for 2-MIB, suggesting the structures of methylated monoterpene alcohols, while 22 compounds showed like 2-MB a molecular ion at *m*/*z* 150 in the mass spectra, in agreement with the structure of a methylated monoterpene hydrocarbon. The main products 2-MIB and 2-MB were detected in the headspace of all four production strains. Furthermore, the previously described products 2-methyl-2-bornene, 1-methylcamphene, 2-methylmyrcene, 2-methyllimonene, 2-methyl-β-fenchol, 2-methyllinalool, 2-methyl-α-terpineol, 2-methylgeraniol and 2-methylnerol were produced by the different strains in strain-specific proportions. 2-Methyl-α-terpineol (RI = 1291) coelutes with indole, which shows high signals in the total ion chromatograms of all investigated *E*. *coli* strains and has a similar retention index of 1294. However, via ion trace analyses it was possible to identify 2-methyl-α-terpineol as products of the production strains harboring the C11-terpene synthases of *P*. *fluorescens* and *S*. *griseus*.

**Table 3:** C11-compounds detected in the headspace of four C11-terpene production strains (11-p, 11-m, 11-g, 11-c) and one control strain without TS (11–0).

		Detected in culture of strain						
#	Compound^b^	11-p	11-c	11-g	11-m	11–0	Identified by^a^	RI
8	C11-terpene	x			x			958
11	2-methyl-2-bornene	x	x		x		Ref	980
12	1-methylcamphene	x	x	x	x		Ref	986
15	2-methylenebornane	x	x	x	x		Ref	1018
17	C11-terpene				x			1032
19	C11-terpene			x				1041
20	C11-terpene	x						1044
22	C11-terpene				x			1052
24	C11-terpene	x			x			1064
25	C11-terpene			x				1073
26	2-methylmyrcene	x	x	x	x	x	Lit	1081
27	C11-terpene				x			1091
28	C11-terpene				x			1095
30	C11-terpene	x						1099
31	C11-terpene	x		x	x			1115
32	C11-terpene	x	x	x		x		1125
33	2-methyllimonene	x	x	x	x		Ref	1128
35	C11-terpene	x			x			1138
36	C11-terpene	x	x	x	x	x		1139
37	3,4-dimethylcumene	x	x	x	x		Ref	1146
41	C11-terpene	x		x	x			1164
45	2-methylisoborneol	x	x	x	x		Ref	1186
46	2-methyl-β-fenchol		x		x			1189
47	C11-terpene	x		x				1191
48	2-methyllinalool	x	x	x	x	x	Ref	1193
49	C11-terpene	x						1194
50	2-methylborneol				x		Ref	1204
51	C11-terpene alcohol	x						1207
55	C11-terpene alcohol			x				1234
59	C11-terpene alcohol	x						1275
60	2-methyl-α-terpineol	x		x			Ref	1293
61	2-methylcitronellol, diastereomer 1			x			Ref	1306
62	2-methylnerol	x					Ref	1308
63	2-methylcitronellol, diastereomer 2		x	x			Ref	1310
64	2-methylgeraniol	x	x	x	x	x	Ref	1327

^a^ Compounds were identified via comparison of mass spectra and RIs of reference compounds (Ref) or of literature data (Lit, [22]).

^b^ Compounds with unknown structures were named regarding their highest *m/z* value as C11-terpene (*m/z* = 150) or C11-terpene alcohol (*m/z* = 168)

**Fig 3:**
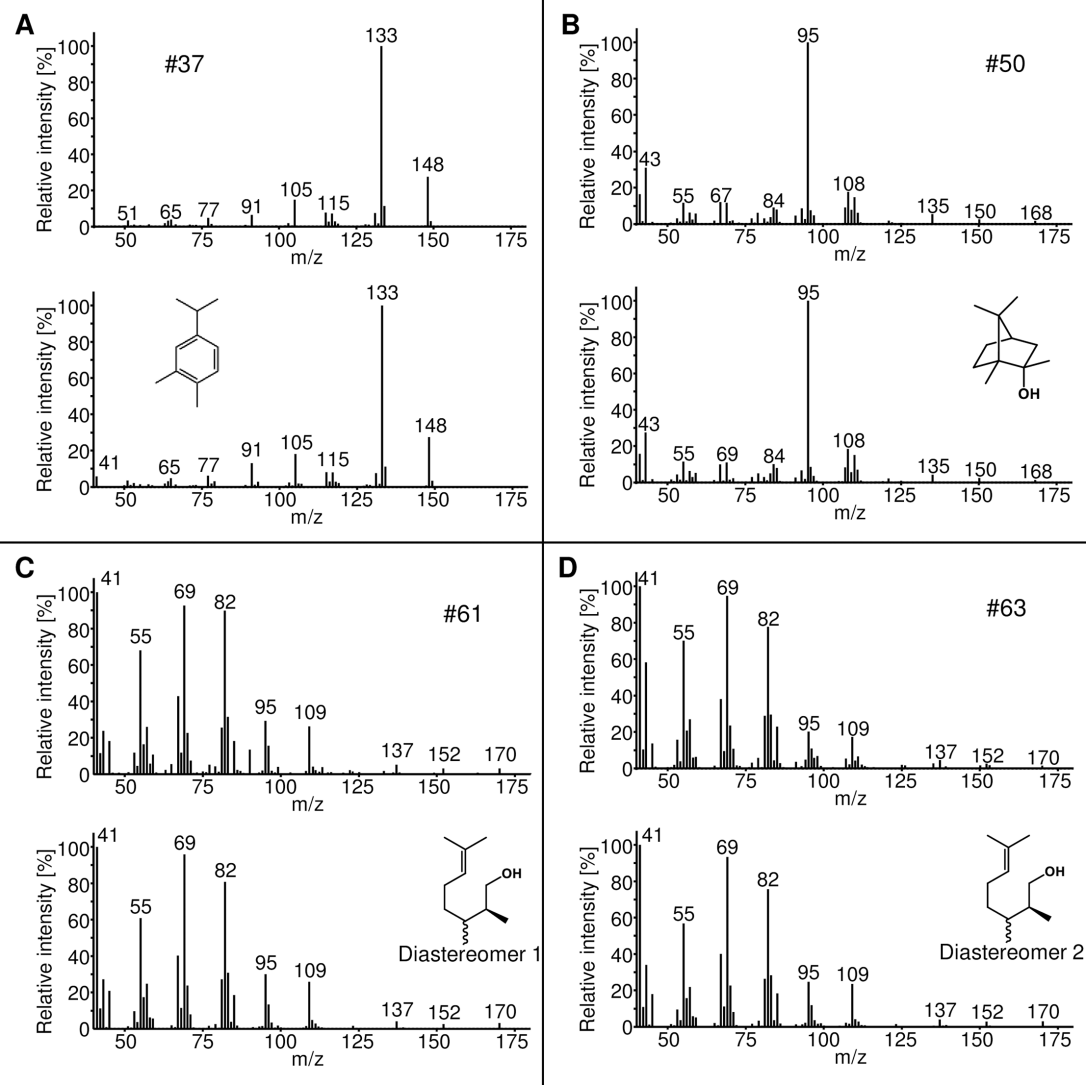
**Mass spectra and structures of the four novel C11-TS products 3,4-dimethylcumene (A), 2-methylborneol (B), 2-methylcitronellol diastereomer 1 (C) and 2-methylcitronellol diastereomer 2 (D)** The mass spectra obtained from the analyses of the production strains (above) are compared with those of reference compounds (below).The mass spectra of the diastereomers of 2-methylcitronellol are almost identical.

Four identified compounds have not been described as C11-TS products or as natural products in general. 2-Methylborneol (*exo*-isomer of 2-MIB), 3,4-dimethylcumene and both diastereomers of 2-methylcitronellol were detected. In contrast to the other identified C11-terpenes, 3,4-dimethylcumene exhibit the molecular ion at *m/z* = 148 and the two diastereomers of 2-methylcitronellol at *m*/*z* = 170 (Fig 3), requiring an additional oxidation step for 3,4-dimethylcumene and a reduction step for 2-methylcitronellol.

In addition to previously described C11-TS products and those four newly found compounds, 20 new C11-terpenes with unknown structures were detected.

From a total of 35 identified C11-terpenes, 2-methylmyrcene, 2-methyllinalool, 2-methylgeraniol and two C11-terpenes with unknown structures are produced by strain 11–0 (Table 3 and S8 Fig), which does not express a terpene synthase. This indicates that they are formed from 2-methyl-GPP without participation of a TS.

### Monoterpenoids

In the headspace of four production strains also 29 monoterpenoids have been detected. We were able to identify hydrocarbons (cylclofenchene, 2-bornene, tricyclene, α-thujene, α- and β-pinene, camphene, sabinene, β-myrcene, α-phellandrene, limonene, (Z)- and (E)-β-ocimene, γ-terpinene, alloocimene), alcohols (linalool, isopulegol, borneol, terpinen-4-ol, γ-isogeraniol, β-citronellol, geraniol) and aldehydes (citronellal, isoneral, isogeranial, neral, geranial). A couple of them only occur in strains expressing one of the C11-TS (S2 Table), showing the acceptance of GPP as substrate of the investigated C11-TS as reported previously [7,34]. However, 16 monoterpenoids are produced as well by strains not expressing any TS, indicating that that they are formed from GPP without participation of a TS.

### Sesquiterpenoids and C16-terpenoids

**Fig 4:**
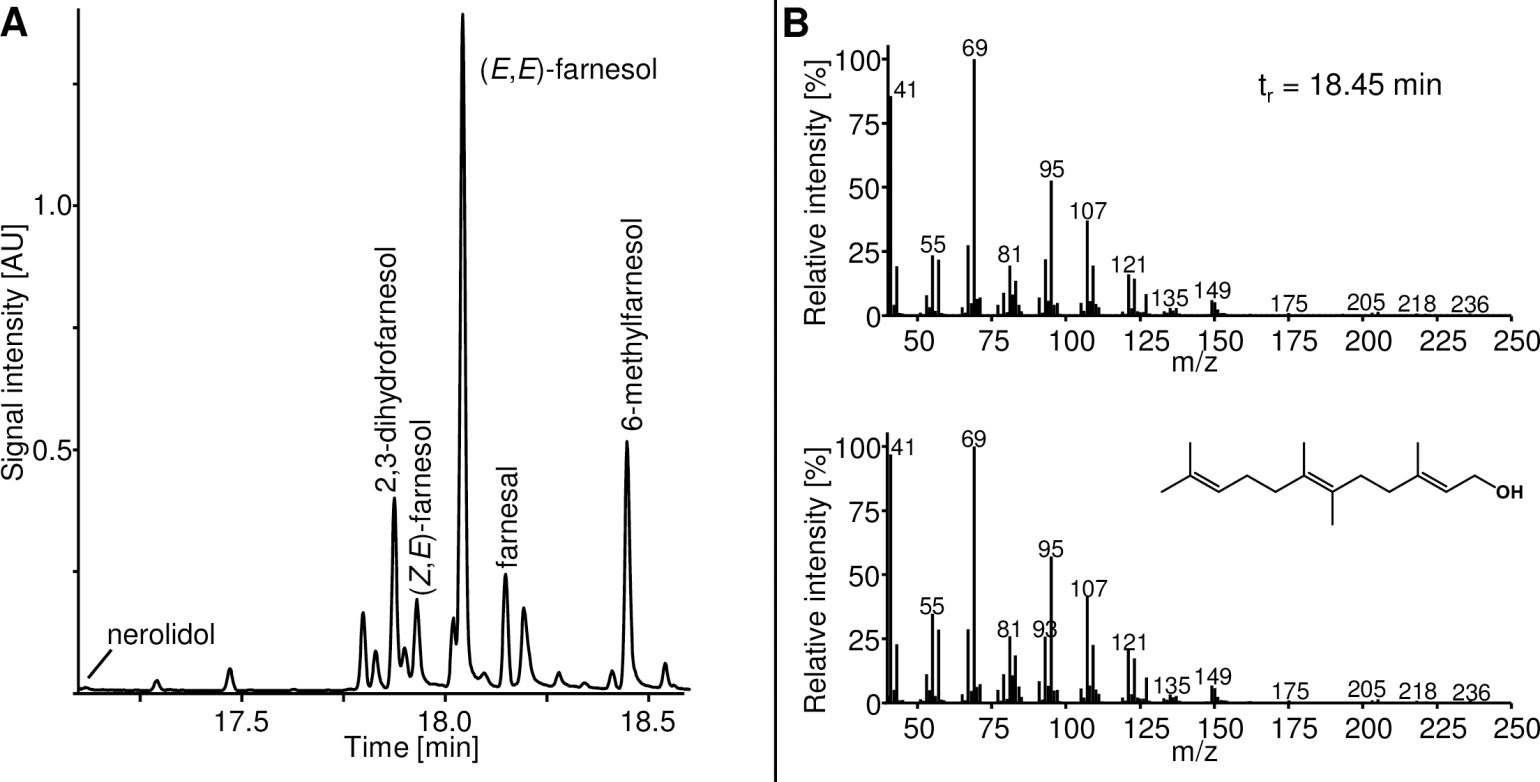
**Total ion chromatogram of SBSE-GCMS analysis of the control strain 11–0 (A) and the structure and mass spectra of 6-methylfarnseol (B)** The spectra of the 6-methylfarnesol peak of the shown chromatogram (above) is compared with that of the reference compound (below).

The formation of less volatile terpenoids by the production and the control strains was investigated using SBSE GC-MS analyses. With nerolidol, 2,3-dihydrofarnesol, (*Z*,*E*)-farnesol, (*E*,*E*)-farnesol and farnesal, five sesquiterpenoids were identified by comparison of their mass spectra to known spectral data and RIs. Furthermore, the production of 6-methylfarnesol could be shown by comparison of the mass spectrum and the RI to the ones of the reference substance (Fig 4).

**Fig 5:**
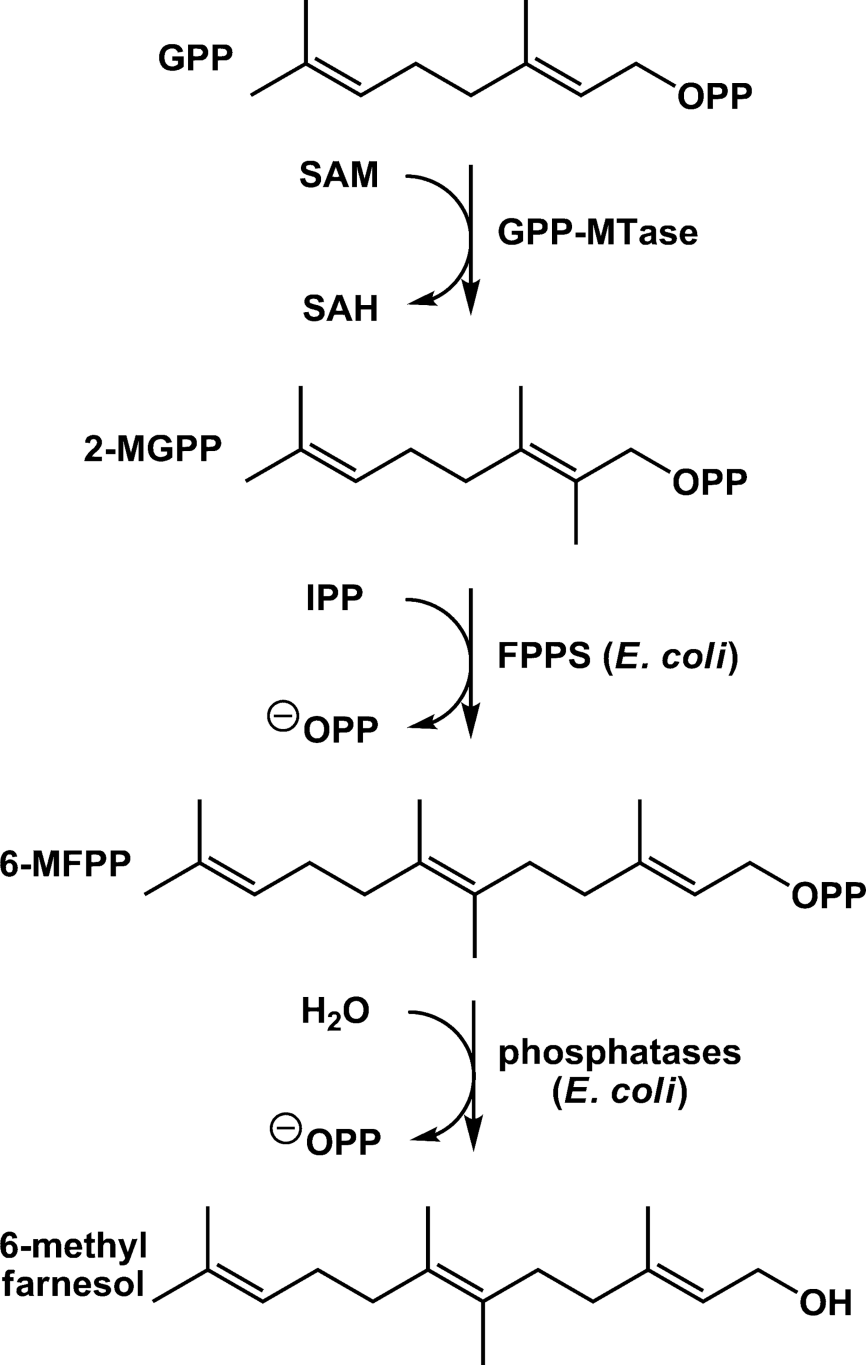
Proposed biosynthesis of 6-methylfarnesol in *E*.*coli* expressing the GPP-MTase of *S*. *coelicolor*. The elongation of 2-MGPP with IPP by an endogenous FPP synthase (FPPS) to 6-methylfarnesyldiphosphate (6-MFPP) and its dephosphorolation by endogenous phosphatases (probably PgpB and YbjG) are assumed.

Like the sesquiterpenoids, this C16-terpene was produced by the production strains and 11–0 indicating that the TS is not involved in the formation of these compounds. Though the control strain 10–0 without the GPP-MTase and TS produced the sesquiterpenoids, it did not produce 6-methylfarnesol, revealing the participation of the GPP-MTase in the production of 6-methyfarnesol. Presumably, the endogenous *E*. *coli* farnesyl diphosphate (FPP) synthase, encoded by *ispA*, catalyzes the condensation of 2-methyl-GPP with IPP to 6-methyl-FPP. This is probably dephosphorylated or hydrolyzed to 6-methylfarnesol in the next step, either spontaneously or by another endogenous enzyme (Fig 5).

## Discussion

After the elucidation of the biosynthetic pathway of 2-MIB and 2-MB [5–7] and the description of eleven C11-terpenes as products of specific bacterial strains or C11-TSs [20,22,23], we could produce 35 different C11-terpenoids via a *de novo* approach and heterologous expression with *E*. *coli*. This enormous product variety originating from only four different C11-TSs reveals that there is a high potential to discover even more C11-terpenes by investigating other C11-TSs or through protein engineering.

Besides the eleven previously described C11-terpenes, 24 novel compounds could be detected in the headspace of four different production strains. From those, the structure of four substances could be identified. Thereby, 3,4-dimethylcumene and the two diastereomers of 2-methylcitronellol are downstream metabolites of further conversions of primary C11-terpene synthase products that are formed by spontaneous or enzymatically catalyzed reactions. 3,4-Dimethylcumene is likely the product of a spontaneous oxidation of 2-methyl-γ-terpinene, in analogy to the reported spontaneous oxidation of the corresponding monoterpene γ-terpinene to p-cymene [35,36]. Since no reference substance is available for 2-methyl-γ-terpinene the presence of the probable precursor of 3,4-dimethylcumene could not be proven in this study.

**Fig 6:**
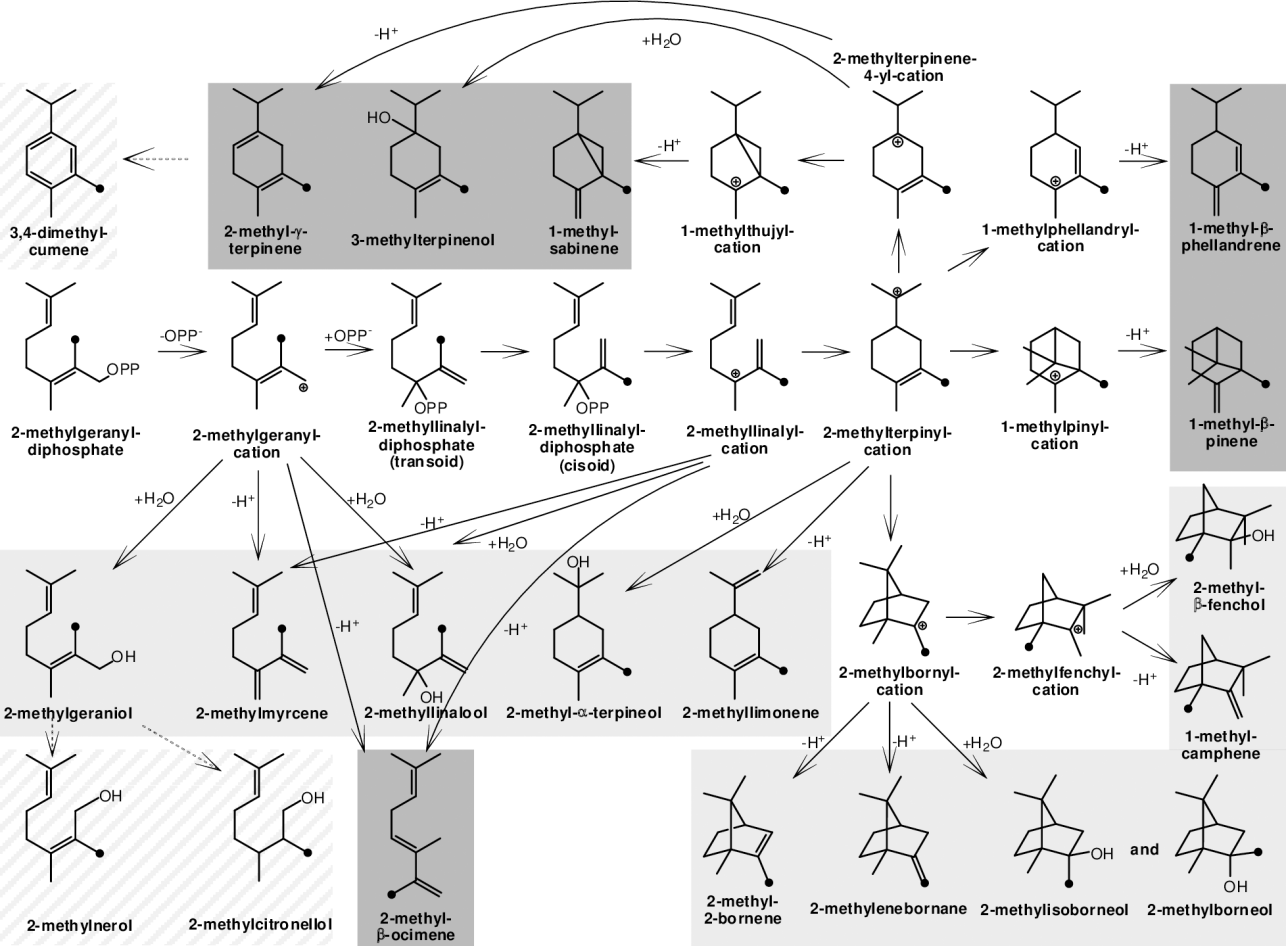
Proposed reaction mechanism of C11-terpenes catalyzed by C11-TSs based on Brock et al. [22]. Methyl groups introduced by the GPP-MTase are labeled with a black dot. Potential, but not detected products have a dark gray background. Products detected in this or other studies have a light gray background. Compounds that have been detected, but are no direct terpene synthase products have a hatched background.

2-Methylcitronellol is probably produced by hydrogenation of 2-methylgeraniol. A spontaneous reaction is unlikely, but in *Saccharomyces cerevisiae*, for example, the reduction of geraniol to citronellol by endogenous enzymes has been described [37]. The proposed reaction mechanisms of described and potential C11-terpenes as well as the suggested subsequent reactions are shown in Fig 6.

The phenomenon of the occurrence of various monoterpenoids in *E*. *coli* strains producing a high level of GPP has already been demonstrated [38]. Fischer et al. showed that different monoterpenes like nerol, linalool and even the cyclic α-terpineol can derive from GPP or geraniol, when GPP is overproduced in *Saccharomyces cerevisae*. Furthermore, the *E*. *coli* enzyme YjgB is known to convert geraniol to nerol, geranial and neral [39]. These findings provide an explanation for the variety of 16 monoterpenes that occur in all strains harboring the pMK-05 plasmid, even in those that do not express any terpene synthase. Nevertheless, we could unequivocally establish that the studied C11-TSs produce further monoterpenes from GPP.

A highly interesting additional product of *E*. *coli* strains expressing the GPP-MTase is the C16-terpene 6-methylfarnesol. Previous studies showed that different prenyltransferases accept various substrate analogs. For example, pig liver FPP synthase can convert various methylated GPP derivatives and C6 homologs of IPP [40], while undecaprenyl diphosphate synthase from *Micrococcus luteus* accepts the C6 compound 3-ethylbut-3-enyl diphosphate and also the C4 analog of IPP, but-3-enyl diphosphate, for the elongation of FPP [41]. Therefore, it can be assumed that 6-methyl-FPP is derived from elongation of the GPP-MTase product 2-methyl-GPP with IPP by the *E*. *coli* FPP synthase encoded by *ispA*. Dephosphorylation of 6-methyl-FPP to 6-methylfarnesol could be a spontaneous reaction or catalyzed by an endogenous phosphatase, e. g. by one of the two integral membrane phosphatases PgpB and YbjG that are known to hydrolyze FPP to farnesol [42].

Our study provides a detailed view on the product diversity accessible by conversion of 2-methyl-GPP with different C11-terpene synthases and provides a cellular synthesis route for 6-methyl-FPP.

## Supporting information

S1 Table Oligos and RBS sequences used in this study (overhangs of primers are underlined). (PDF)

S2 Table Monoterpenoids detected in the headspace of four C11-terpene production strains (11-p, 11-m, 11-g, 11-c) and one control strain without GPP-MTase and TS (10–0). (PDF)

S1 Fig *gppmtase* sequence optimized in codon usage for *E*. *coli*. (PDF)

S2 Fig *mbsp* sequence optimized in codon usage for *E*. *coli*. (PDF)

S3 Fig *mbsm* sequence optimized in codon usage for *E*. *coli*. (PDF)

S4 Fig *mibsg* sequence optimized in codon usage for *E*. *coli*. (PDF)

S5 Fig *mibsc* sequence optimized in codon usage for *E*. *coli*. (PDF)

S6 Fig Mass spectra of all compounds with unknown structure listed in Table 3 or S2 Table. (PDF)

S7 Fig Mass spectra of C11- and C16-reference substances. (PDF)

S8 Fig Total ion chromatograms of HS-SPME-GCMS analyses of the control strains 0–0, 10–0 and 11–0 compared to the one of the production strain 11-p. (PDF)
