# Supplementary material for: Heterologous expression of 2-methylisoborneol / 2 methylenebornane biosynthesis genes in Escherichia coli yields novel C11-terpenes
Source: PLoS One. 2018 Apr 19;13(4):e0196082. doi: 10.1371/journal.pone.0196082 (PMC5908152; doi:10.1371/journal.pone.0196082)
Supplement: S1 Table — (PDF) [file pone.0196082.s001.pdf]

S1 Table. Oligos and RBS sequences used in this study (overhangs of primers are underlined)

Name	Sequence
mk20	GAGCTCGCTTGGACTCCT
mk23	TTAATTAAGACGTCGGTGCCTAATGAGT
mk24	ACAGGAGTCCAAGCGAGCTCGCGGCGTTGTGACAATTTACC
mk25	ACTCATTAGGCACCGACGTCTTAATTAAGGACGCACACCGTGGAAA
mk7	CCTTAATTCTGACGAAATGCC
mk8	CCTAAGGATCCAAACTCGAGTAA
mk78x	GCATTTCGTCAGAATTAAGGCCTAAGGATCCAAACTCGAG
mk26	AGCTGGATCCTCCTAAGATCTTTTGAATTCTGAAATT
mk10	TTAGTGTCGTCTTAACTTTAGACTAGTCCCGGGTGGGGAATTGTTATCCGCTC
mk11	ACCCTCGAGTCTGGTAAAGA
mk12	AGTCTAAAGTTAAGACGACACTAAGGAGGATATAGATGACCACCGAAACCACCAC
mk13	TCTTTACCAGACTCGAGGGTTTACACACGATCTGCGGCAA
RBS (*mbsp*)	CGAAACTAATCGCAGTATAATAAGGAGGTAAGTC
RBS (*mbsm*)	AGAAGAACACGGGGTCAATAAGGAGGTTAATT
RBS (*mibsg*)	CGAGAGACGCGCTCGCCATTTAAGGAGGGTCTTC
RBS (*mibsc*)	AATTCAGTAAACAGCATAAGGAGAGTAACAT
RBS (*gppmtase*)	CTAAAGTTAAGACGACACTAAGGAGGATATAG
