# Supplementary material for: Heterologous expression of 2-methylisoborneol / 2 methylenebornane biosynthesis genes in Escherichia coli yields novel C11-terpenes
Source: PLoS One. 2018 Apr 19;13(4):e0196082. doi: 10.1371/journal.pone.0196082 (PMC5908152; doi:10.1371/journal.pone.0196082)
Supplement: S2 Table — (PDF) [file pone.0196082.s002.pdf]

S2 Table. Monoterpenoids detected in the headspace of four C11-terpene production strains (11-p, 11-m, 11-g, 11-c) and one control strain without GPP-MTase and TS (10-0)

#	Compound[b]	Detected in culture of strain					Identified by[a]	RI
		11-p	11-c	11-g	11-m	10-0		
1	cyclofenchene	x					SI, RI	882
2	2-bornene	x					SI, RI	906
3	tricyclene	x					SI, RI	923
4	α-thujene	x					SI, RI	928
5	α-pinene	x			x		Ref	934
6	monoterpene	x						940
7	camphene	x		x			SI, RI	950
9	sabinene	x			x		SI, RI	974
10	β-pinene	x			x		Ref	977
13	β-myrcene	x	x	x	x	x	Ref	991
14	α-phellandrene	x	x			x	Ref	1009
16	limonene	x	x	x	x	x	Ref	1031
18	(Z)-β-ocimene	x	x			x	Ref	1038
21	(E)-β-ocimene	x	x			x	SI, RI	1049
23	γ-terpinene	x	x				Ref	1060
29	linalool		x	x		x	Ref	1099
34	alloocimene					x	Ref	1129
38	isopulegol		x			x	Ref	1149
39	citronellal	x	x	x		x	Ref	1151
40	isoneral		x			x	SI, RI	1162
42	borneol	x					SI, RI	1170
43	terpinen-4-ol	x					Ref	1180
44	isogeranial		x			x	SI, RI	1180
52	γ-isogeraniol	x	x				SI, RI	1218
53	β-citronellol	x	x	x	x	x	Ref	1227
54	(Z)-isogeraniol	x	x			x	SI RI	1232
56	neral	x	x	x		x	Ref	1241
57	geraniol	x	x	x		x	Ref	1254
58	geranial	x	x	x		x	Ref	1270

[a] Compounds were identified via comparison of mass spectra and RIs of reference compounds (Ref) or mass spectra of the NIST mass spectral library (v14) and RIs (SI, RI) published by Adams (29).

[b] Compounds with unknown structures were named regarding their highest *m/z* value as monoterpene (*m/z* = 136) or monoterpene alcohol (*m/z* = 154).
