# Supplementary material for: Heterologous expression of 2-methylisoborneol / 2 methylenebornane biosynthesis genes in Escherichia coli yields novel C11-terpenes
Source: PLoS One. 2018 Apr 19;13(4):e0196082. doi: 10.1371/journal.pone.0196082 (PMC5908152; doi:10.1371/journal.pone.0196082)
Supplement: S1 Fig — (PDF) [file pone.0196082.s003.pdf]

S1 Fig. *gppmtase* sequence optimized in codon usage for *E. coli*

```
1    ATGACCACCGAAACCACCAC CGCAACCGCGACCGCGAAAA TTCCTGCTCCGGCAACCCCG TATCAAGAAGATATTGCACG TTATTGGAATAATGAAGCCC
101  GTCCGGTTAATCTGCGTCTG GGTGATGTTGATGGCCTGTA TCACCATCATTATGGTATTG GTCCGGTTGATCGTGCAGCC CTGGGTGATCCTGAACATTC
201  AGAATATGAAAAAAAAGTGA TCGCCGAACTGCATCGTCTG GAAAGCGCACAGGCAGAATT TCTGATGGATCATCTGGGTC AGGCAGGTCCGGATGATACC
301  CTGGTTGATGCAGGTTGTGG TCGTGGTGGTAGCATGGTTA TGGCACATCGTCGTTTTGGT AGCCGTGTGGAAGGTGTTAC CCTGAGCGCAGCACAGGCCG
401  ATTTTGGTAATCGTCGTGCA CGTGAACTGCGTATTGATGA TCATGTTCGTAGTCGTGTTT GCAATATGCTGGATACCCCG TTTGATAAAGGTGCAGTTAC
501  CGCAAGCTGGAATAACGAAA GCACCATGTATGTTGACCTG CATGACCTGTTTAGCGAACA TAGCCGTTTTCTGAAAGTTG GTGGTCGCTATGTTACCATT
601  ACAGGTTGTTGGAATCCGCG TTATGGCCAGCCGAGCAAAT GGGTTAGCCAGATTAATGCA CATTTTGAGTGCAACATTCA TAGCCGTCGCGAATATCTGC
701  GTGCAATGGCAGATAATCGT CTGGTTCCGCATACCATTGT TGATCTGACACCGGATACCC TGCCGTATTGGGAACTGCGT GCGACCAGCAGCCTGGTTAC
801  CGGTATTGAAAAAGCATTTA TCGAGAGCTATCGTGATGGC AGCTTTCAGTATGTTCTGAT TGCCGCAGATCGTGTGTAA
```
