# Supplementary material for: Heterologous expression of 2-methylisoborneol / 2 methylenebornane biosynthesis genes in Escherichia coli yields novel C11-terpenes
Source: PLoS One. 2018 Apr 19;13(4):e0196082. doi: 10.1371/journal.pone.0196082 (PMC5908152; doi:10.1371/journal.pone.0196082)
Supplement: S2 Fig — (PDF) [file pone.0196082.s004.pdf]

S3 Fig. *mbsm* sequence optimized in codon usage for *E. coli*

```
1    ATGAGCGCAGCAGATGCACT GAGCGGTTTTGCAGCCGATG CCCTGTCAGGTTTTGCCGCT GATGCGCTGAGTGGCTTTGC CGCAGCAATTCTGGCAGCAA
101  CCGGTCGTGCACCGAGCGCA GAACTGAGCCAGGTTGCAGC AGGTCCGACCGCACTGGATC GTCTGACCGATAGCACCGGT CTGGGTCGTAGCGCATTTCG
201  TATTCCGCGTAGCCCGATGC TGCCTCCGCCTACCGATGAT GGTGTGCCGGAACTGTTTTG TCCGGGTCCGGTTCGTGATG ATCCGGCACTGGGTGAAACC
301  GTTAATGATGGTATTGTTGA ATGGGCAGGTCAGGTTGGTA TTTATCCGGGTCAGCTGGAT CGCCTGCGTGCATATAACTT TGGTCGTCTGATTATGCTGA
401  CCCATCCGGCAACCAATGAT CCTGATCGTCTGCTGGCAGC AGCAAAATGTGTTGTTGCAG AATGGGCAACCGATGATTAT GTTGTTGATGAAGTTAGCCT
501  GGGTGCAGATCCGGCAGTTG TTGGTAGCCGTCTGGCAAAA CTGCATGCCGTTGTTGATCC TGCCCGTCTGCCTGCACGTT ATGCACCGCAGCTGGATGCA
601  TATCGTCGTGATGAACCGAT TGCAACCGCCTTTCGTAGCG CCATGGAACATCTGGCACGT TATACCACCGTTGCACAGCT GGGTCGTTTTCAGCATCAGC
701  TGGGTATTCTGTTTCTGGCA TGGAATCAAGAAGCAGATTG GCACGTAAATGGTCGTACCC CTCCGGTTTGGGAATATCTG GTTCAGCGTCATCTGAATAA
801  CTTTCTGCCTCCGATGGTTC TGGTTGATGCAGTTGCAGGT TATGAACTGAGTCCGGATGA ATTTTTTGATCCGCGTGTTC GTCGTGCCTATACCACAGCA
901  GCACTGGCAAATGTTCTGCT GAATGATATTCATAGCGGCA CCTGTGAAAGCGATACCGAT TTTAATCTGCCTCGTGTGAT TAGCATTGAAGAGGGTTGTA
1001 GCCTGCGTGATGCCGTTACC CGTACCGTTGAAATTCATAA TGAACTGATGCATGCGTTTG TTGCCGATGCAGCAACCCTG AGCCTGATTGGTAGCCCGAA
1101 TCTGCGTCGTTTTCTGGCAG ATATTTGGGCATGGCTGGGT GGTAGTCGTGAATGGCATGC AACCACCAGTCGTTATCATG GTGAAGCAACCACCCGTAGC TAA
```
