# Supplementary material for: Heterologous expression of 2-methylisoborneol / 2 methylenebornane biosynthesis genes in Escherichia coli yields novel C11-terpenes
Source: PLoS One. 2018 Apr 19;13(4):e0196082. doi: 10.1371/journal.pone.0196082 (PMC5908152; doi:10.1371/journal.pone.0196082)
Supplement: S3 Fig — (PDF) [file pone.0196082.s005.pdf]

S2 Fig. *mbsp* sequence optimized in codon usage for *E. coli*

```
1    ATGAATCAGAGCAGCAGCGC ACGTACACCGCGTAGCGCAA CCGCACCGTTTATTGTTCGT GCAGTTCGTTGTCCGCCTCC GACCCGTATTGATGAAGCAC
101  TGGGTCAAGAAGTTAACGAA CGTCTGATGGAATGGATTAG CAACATTGGTATTTTTGCCG GTAAAGAAGAGAAAATTCGC GCAAGCGATTTTGGTCGTTA
201  TGCAATGCTGTGTCATGCCG ATACCAATAATCCGGATCGT CTGCTGCTGGTTGCACAGTG TTTTGCAGCACTGTTTGCAG TTGATGATCATTATTGTGAT
301  GATCAGAGCCTGGGTGGTCG TCCGGAAACCGTTGCAGAAA GCCTGAGCTTTGCACTGACC GCAATTGATCCGGTTTATCT GCCGAGCCCGTTTGATAAAG
401  AACTGCTGAAACAGCAGATG TGTGATCCGGTGATTCGTGG TCTGCTGGCATATATGAAAC GTGTTGCCCAGTTTTGTACC CCGAGCCAGGTTGCCCGTGT
501  TCGTCAGATTACCATTGCCA TGTTTGTTACCATGGCAGCC GAAGGTCCGTGGCGTCTGTA TGGCACCCAGCCGACCGTTG CCGAATATCTGGCAAGCCGT
601  CAGGTTAATAGCTTTTGGCC GTGTCTGGTTCTGATTGATC TGATTGGTGGTTATGAAGTT CCGGCAAATACCTATAGCCG TCCGGATATTCATCATGTTA
701  CCGCACTGGCCAGCCTGGCA ACCACCCTGGTTAATGATCT GTATAGCGCATATAAAGAGC ACCTGAACGAAACCGGTGAT TTTAAACTGCCGTACCTGCT
801  GGCAGCACGTCATAATTGTA GCCTGCAAGAAGCCATTGAT CTGGCAGCAGATATTCATGA TGCAGTGATGGAAGAATATG AACGTCTGCATGCAACCCTG
901  ATGAAAGGCACCCGTAGTCC GGTTCTGCGTCGTTATCTGA CCGGTCTGAGCACCTGGATT GGTGGCAATCTGGAATGGCA TAAACATAGCGCACGCTATC ATATCTAA
```
