# Supplementary material for: Heterologous expression of 2-methylisoborneol / 2 methylenebornane biosynthesis genes in Escherichia coli yields novel C11-terpenes
Source: PLoS One. 2018 Apr 19;13(4):e0196082. doi: 10.1371/journal.pone.0196082 (PMC5908152; doi:10.1371/journal.pone.0196082)
Supplement: S4 Fig — (PDF) [file pone.0196082.s006.pdf]

S4 Fig. *mibsg* sequence optimized in codon usage for *E. coli*

```
1    ATGCCGGTGCCGGAACTGCC TCCGCCTCGTAGCAGTCTGC CGGAAGCAGTTACCCGTTTT GGTGCAAGCGTTCTGGGTGC AGTTGCAGCACGTGCACATG
101  ATAGCGAAGCAACCGTTGGT GGTCCGAGCGGTGGTCGTCC GCTGCCTAGTCCTCCGGCAG GTCTGAGTTTTGGTCCGCCT AGTCCGGCAGCACCGAGCGC
201  AGATGTTCCAGCACCGGAAG CACCGGGTCGTGGTGCAGAT CTGGAACGTCTGCTGTGTGG TCCGCATGGTCTGGGCACCG CAGGTCTGCGTCTGACACCG
301  GGTAAAGAACGTCCGGTTCC GGCAACCGCACGTGAAGGTC GTCCGATTCCGGGTCTGTAT CATCATCCGGTGCCTGAACC GGATGAAGCACGTGTTGAAG
401  AAGTTAGCCGTCGTATTAAA GCATGGGCACTGGATGAAGT GAGCCTGTATCCGGAAGAAT GGGAAGAACAGTTTGATGGT TTTAGCGTTGGTCGTTATAT
501  GGTTGGTTGTCATCCTGATG CACCGACCGTTGATCATCTG ATGCTGGCAACCCGTCTGAT GGTTGCAGAAAATGCAGTTG ATGATTGCTATTGCGAAGAT
601  CATGGTGGTAGTCCGGTTGG TCTGGGTGAACGCCTGCTGC TGGCACATACCGCACTGGAT CCGCTGTATACCGCACGCGA ATATCAGCCTGGTTGGGCAG
701  CAAGCCTGCATGCCGATGCA CCGCGTCGTGCATATCGTAG CGCAATGGATTATTTTGTTC GTGCAGCAGGTCCGAGCCAG GCAGATCGTCTGCGTCATGA
801  TATGGCACGTCTGCATCTGG GTTATCTGGCAGAAGCAGCC TGGGCTCAGCAGGATCAGGT TCCGGAAGTTTGGGAATATC TGGCAATGCGTCAGTTTAAT
901  AACTTTCGTCCGTGTCCGAC CATTACCGATACCGTGGGTG GTTATGAACTGCCTGCCGAT CTGCATGCACAGGCAGCAAT GCAGAAAGTGATTGCACTGG
1001 CAAGTAATGCAACCACCATT GTGAATGATCTGTACAGCTA TACCAAAGAACTGGCAGCTC CGGGTCGTCATCTGAATCTG CCGGTTGTTATTGCAGAACG
1101 CGAAGGTCTGAGCGATCAGG ATGCATATCTGAAAAGCGTG GAAATTCATAACGAACTGAT GCATGCCTTTGAAAGCGAAG CAGCAGCACTGGCAGCAGCA
1201 TGTCCGGTGCCGAGCGTTCA GCGTTTTCTGCGTGGTGTTG CAGCATGGGTTGATGGTAAT CATCATTGGCATCGTAGCAA TACCTATCGTTATAGCCTGC
1301 CGGATTTTTGGTAA
```
