# Supplementary material for: Heterologous expression of 2-methylisoborneol / 2 methylenebornane biosynthesis genes in Escherichia coli yields novel C11-terpenes
Source: PLoS One. 2018 Apr 19;13(4):e0196082. doi: 10.1371/journal.pone.0196082 (PMC5908152; doi:10.1371/journal.pone.0196082)
Supplement: S5 Fig — (PDF) [file pone.0196082.s007.pdf]

S5 Fig. *mibsc* sequence optimized in codon usage for *E. coli*

```
1    ATGCCGGATAGCGGCACCCT GGGTACACCGCCTCCGGAAC AGGGTCCGACCCCTCCGACA ACCCTGCCGGATGTTCCGGC ACCGGTTATTCCGAGCGCAA
101  GCGTTACCAGCGCAGCAAGC GATTTTCTGGCAGCACTGCA TCCGCCTGTTACCGTTCCGG ATCCTGCACCTCCGCCTCCG CCTGCACCGGCAGCAGGTAA
201  TCCTCCGGATACCGTTACCG GTGATAGCGTTCTGCAGCGT ATTCTGCGTGGTCCGACCGG TCCGGGTACAACCAGTCTGG CTCCGGCAGTTCGTTATGGT
301  CGTCAGCCTGGTCCGGAAGC ACCGGCAAGTGCACCGCCTG CAGCAGGTCGTGCAGTTCCG GGTCTGTATCATCATCCGGT GCCTGAACCTGATCCGGTTC
401  GTGTTGAAGAAGTTAGCCGT CGTATTAAACGTTGGGCAGA AGATGAAGTTCAGCTGTATC CGGAAGAATGGGAAGGTCAG TTTGATGGTTTTAGCGTTGG
501  TCGTTATATGGTTGGTTGTC ATCCTGATGCACCGACCGTT GATCATCTGATGCTGGCAAC CCGTCTGATGGTTGCAGAAA ATGCAGTTGATGATTGCTAT
601  TGCGAAGATCATGGTGGTAG TCCGGTTGGTCTGGGTGGTC GTCTGCTGCTGGCACATACC GCAATTGATCATTTTCATAG CACCGCAGAATATACCCCGA
701  CCTGGCAGGCAAGCCTGGCA GCAGATGCACCGCGTCGTGC ATATGATAGCGCAATGGGTT ATTTTGTTCGTGCAGCAACC CCGAGCCAGAGCGATCGTTA
801  TCGTCATGATATGGCACGTC TGCATCTGGGTTATCTGGCA GAAGGTGCCTGGGCACAGAC CGGTCATGTTCCGGAAGTTT GGGAATATCTGGCAATGCGT
901  CAGTTTAATAACTTTCGTCC GTGTCCGACCATTACCGATA CCGTTGGTGGTTATGAACTG CCTGCCGATCTGCATGCACG TCCGGATATGCAGCGTGTTA
1001 TTGCACTGGCAGGTAATGCA ACCACCATTGTTAATGATCT GTACAGCTATACCAAAGAGC TGAATAGTCCGGGTCGTCAT CTGAATCTGCCGGTTGTTAT
1101 TGCCGAACGTGAACAGCTGT GTGAACGTGATGCATATCTG AAAGCAGTTGAAGTGCATAA TGAACTGCAGCATAGCTTTG AAGCAGCAGCAGCCGATCTG
1201 GCCGAAGCATGTCCGCTGCC TCCGGTTCTGCGTTTCCTGC GTGGTGTTGCAGCATGGGTT GATGGTAACCATGATTGGCA TCGTACCAATACCTATCGTT
1301 ATAGTCTGCCGGATTTTTGG TAA
```
