# Supplementary material for: Heterologous expression of 2-methylisoborneol / 2 methylenebornane biosynthesis genes in Escherichia coli yields novel C11-terpenes
Source: PLoS One. 2018 Apr 19;13(4):e0196082. doi: 10.1371/journal.pone.0196082 (PMC5908152; doi:10.1371/journal.pone.0196082)
Supplement: S6 Fig — (PDF) [file pone.0196082.s008.pdf]

S6 Fig. Mass spectra of all compounds with unknown structure listed in Table 3 or S2 Table

Compound: #8

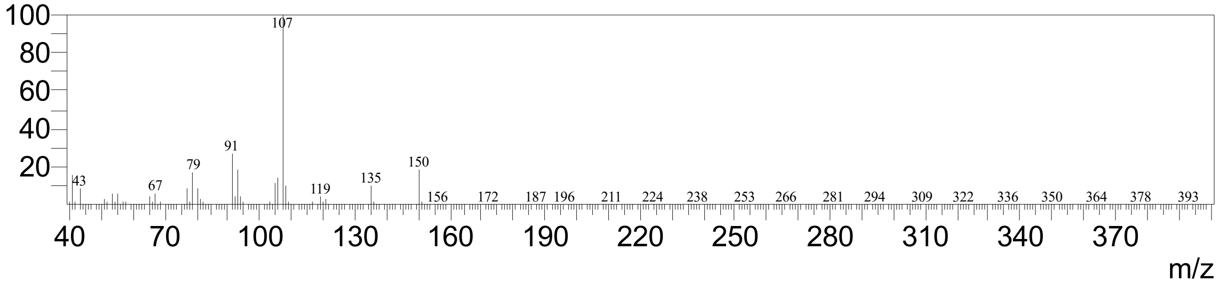


Compound: #17

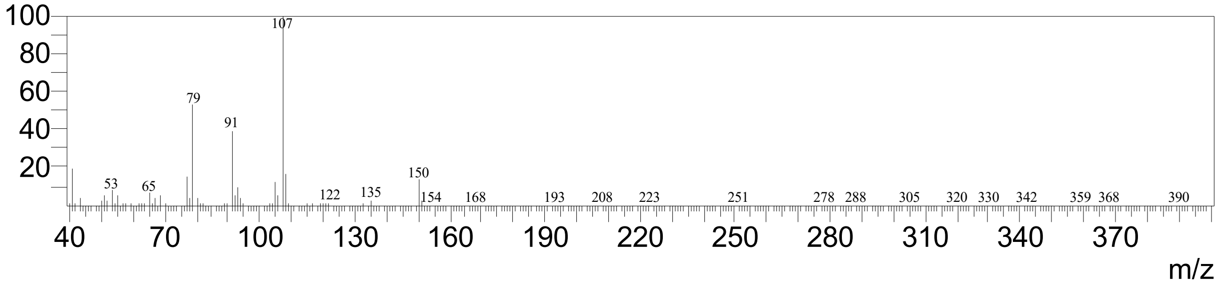


Compound: #19

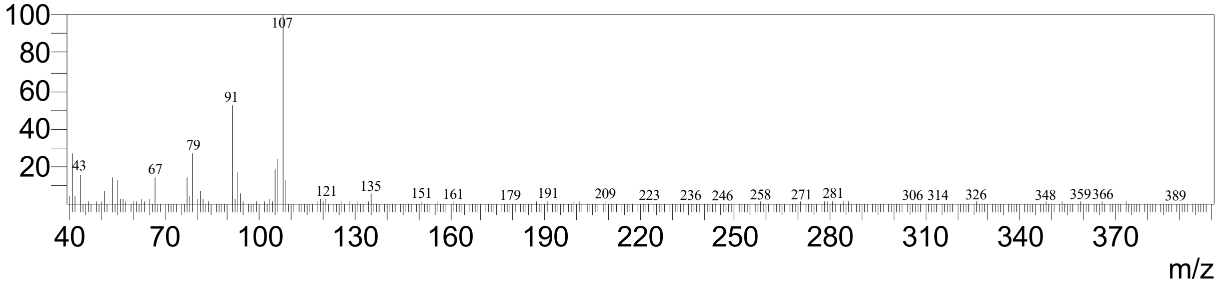


Compound: #20

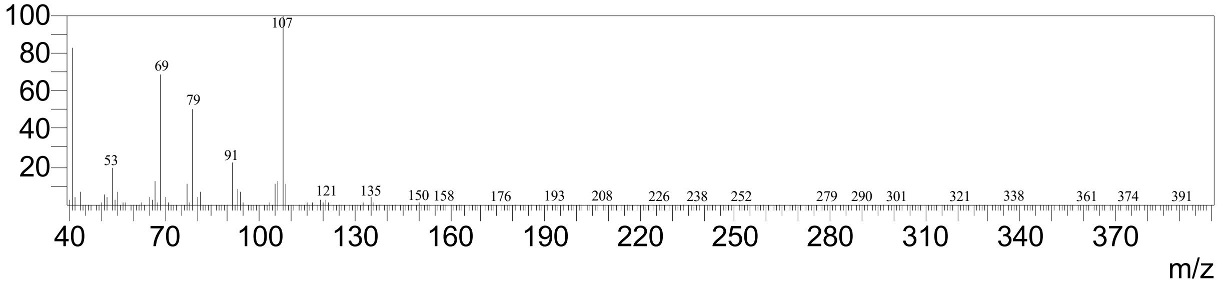


Compound: #22

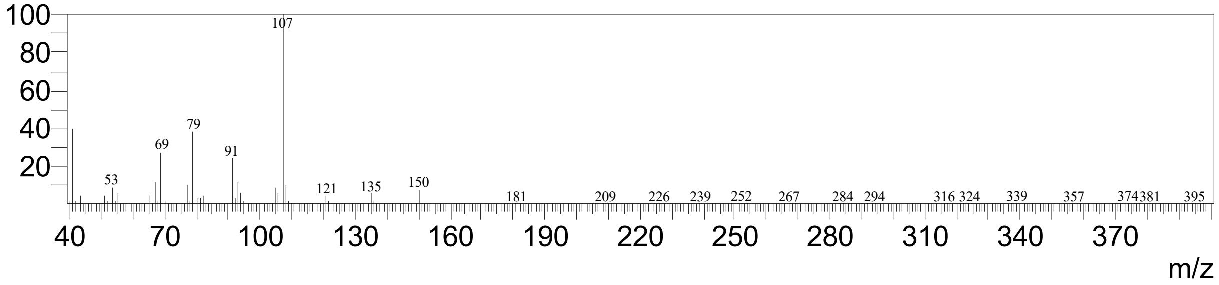

Compound: #24

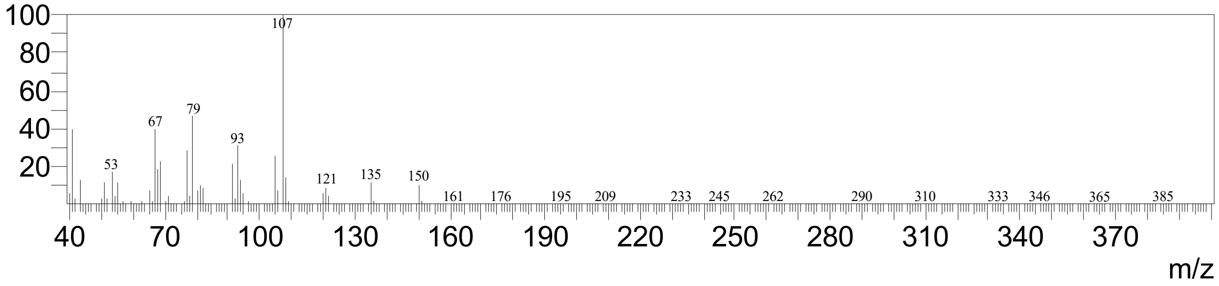


Compound: #25

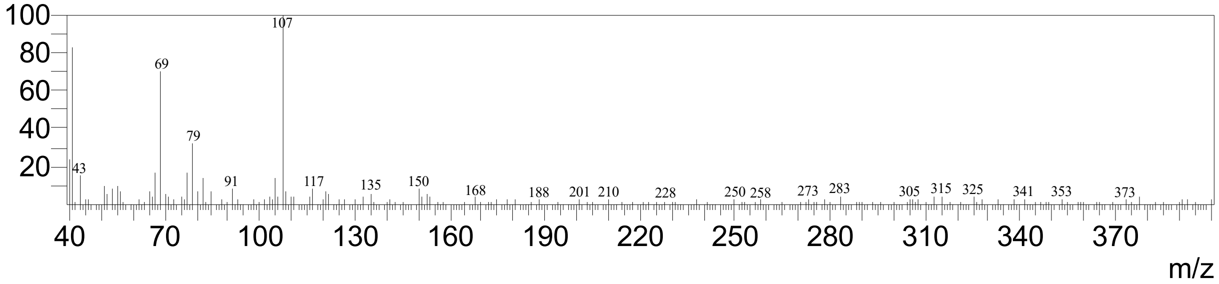


Compound: #27

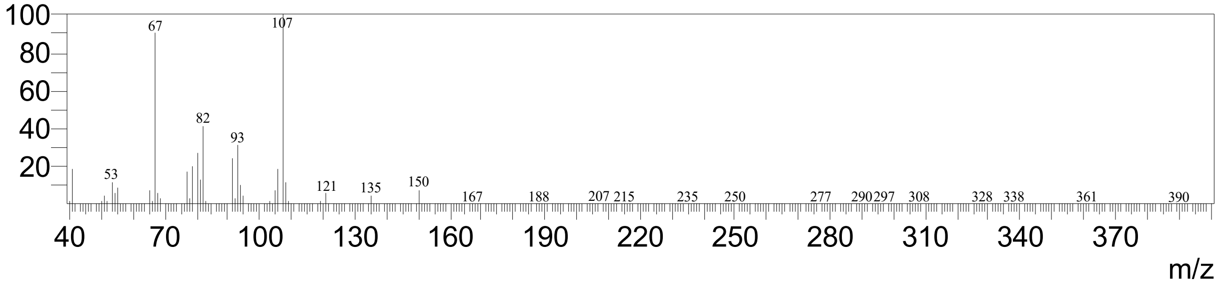


Compound: #28

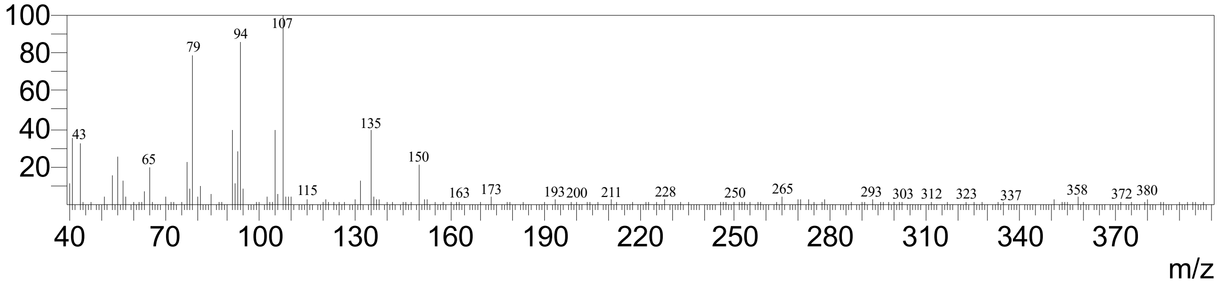


Compound: #30

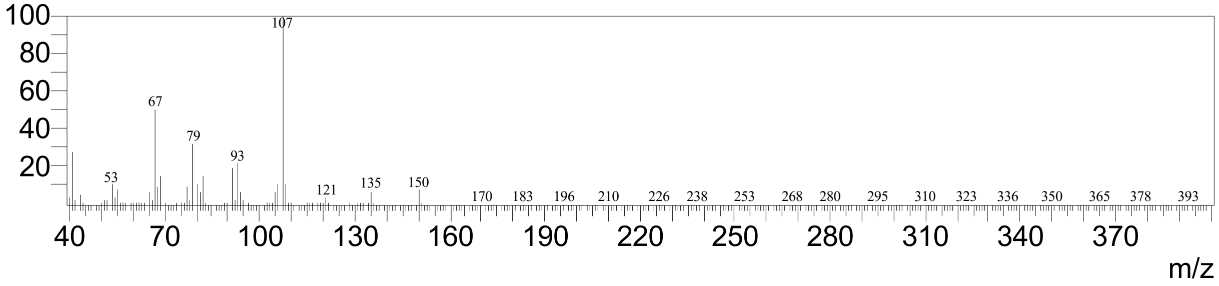

Compound: #31

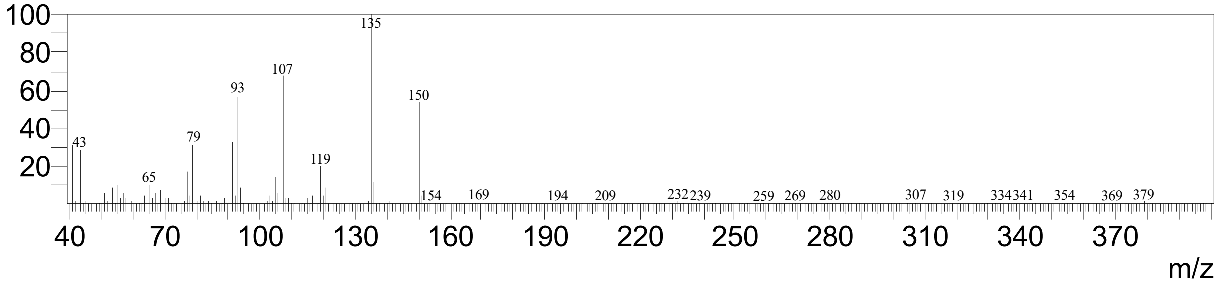


Compound: #32

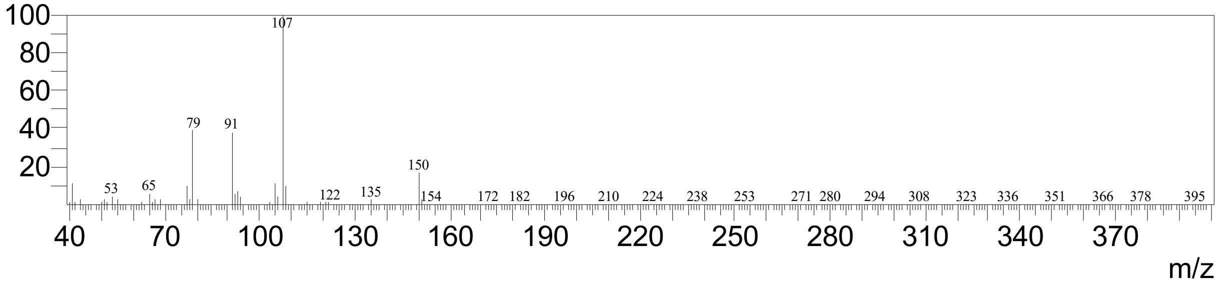


Compound: #35

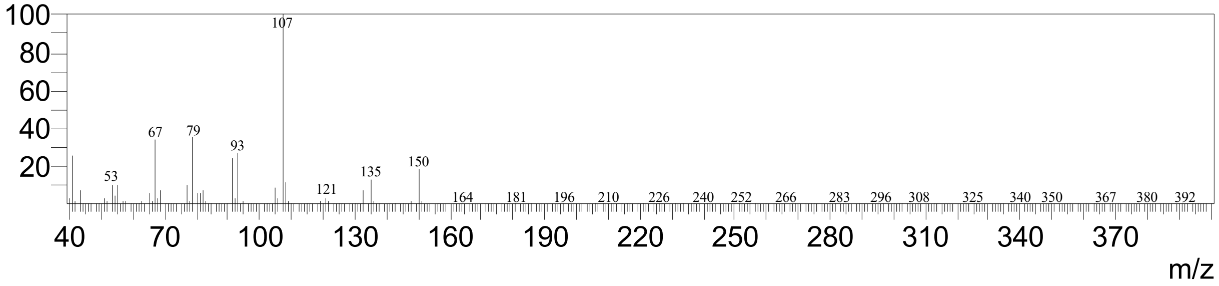


Compound: #36

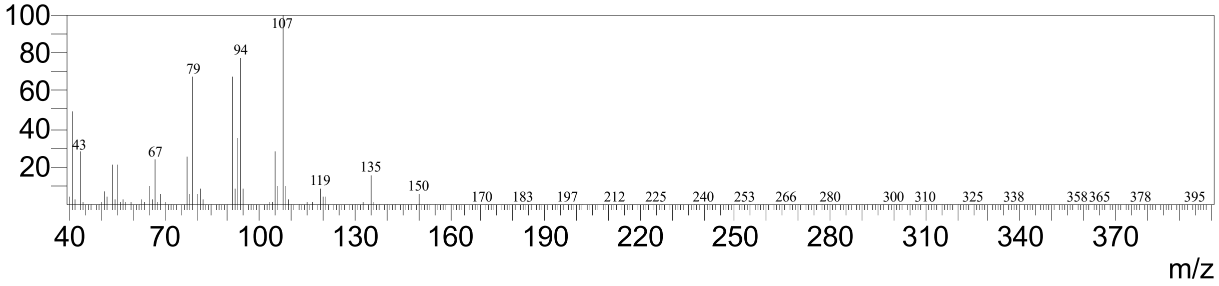


Compound: #41

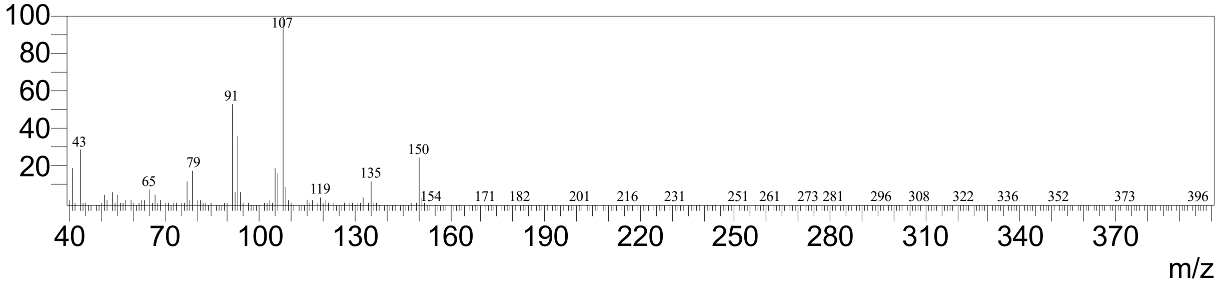

Compound: #47

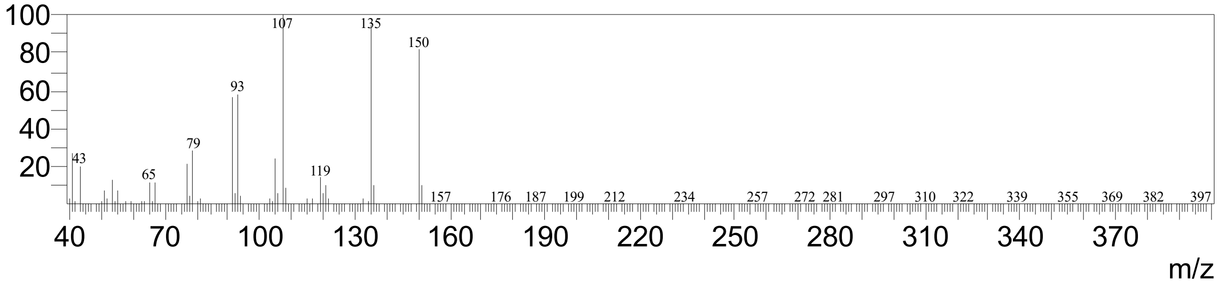


Compound: #49

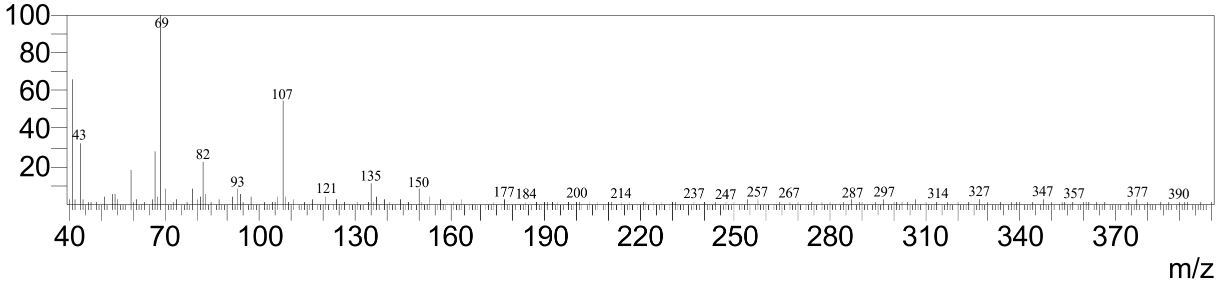


Compound: #51

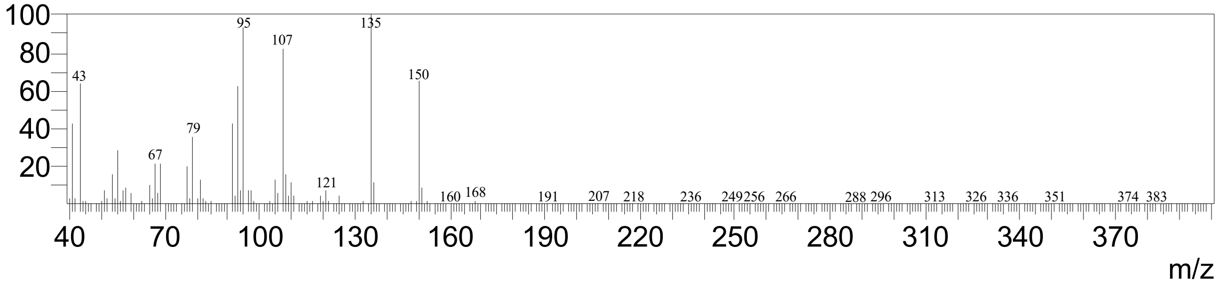


Compound: #55

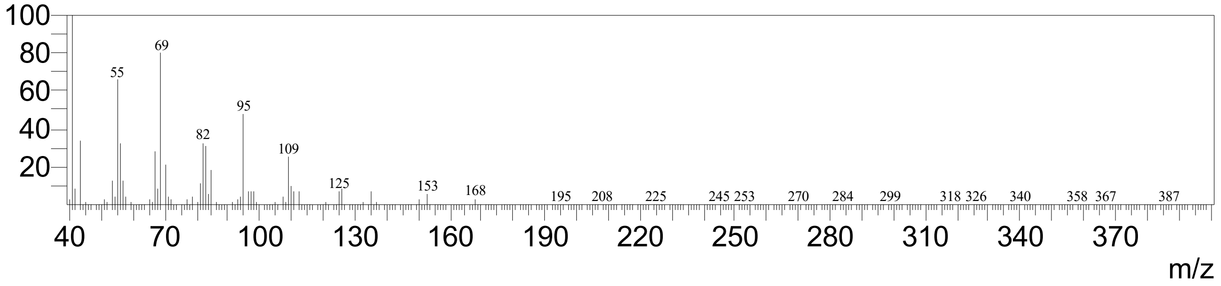


Compound: #59

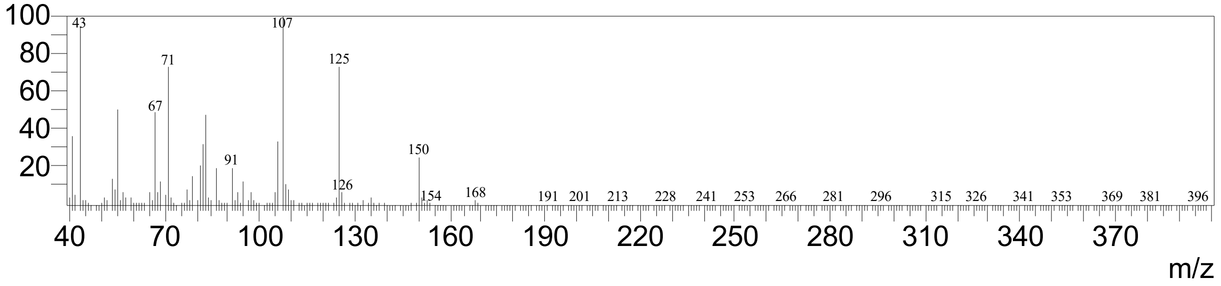

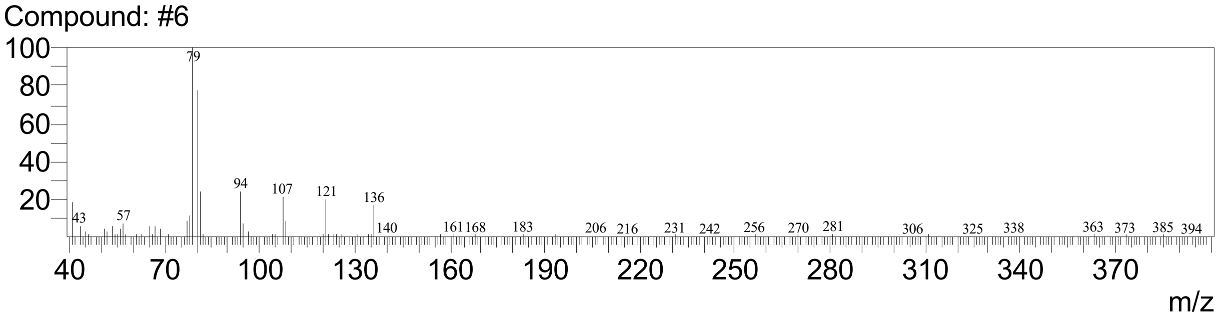

Compound: #6
100
80
60
40
20
79
94
107
121
136
43
57
140
161
168
183
206
216
231
242
256
270
281
306
325
338
363
373
385
394
40
70
100
130
160
190
220
250
280
310
340
370
m/z
