# Supplementary material for: Heterologous expression of 2-methylisoborneol / 2 methylenebornane biosynthesis genes in Escherichia coli yields novel C11-terpenes
Source: PLoS One. 2018 Apr 19;13(4):e0196082. doi: 10.1371/journal.pone.0196082 (PMC5908152; doi:10.1371/journal.pone.0196082)

S7 Fig. Mass spectra of C11- and C16-reference substances

Compound: 2-Methyl-2-bornene

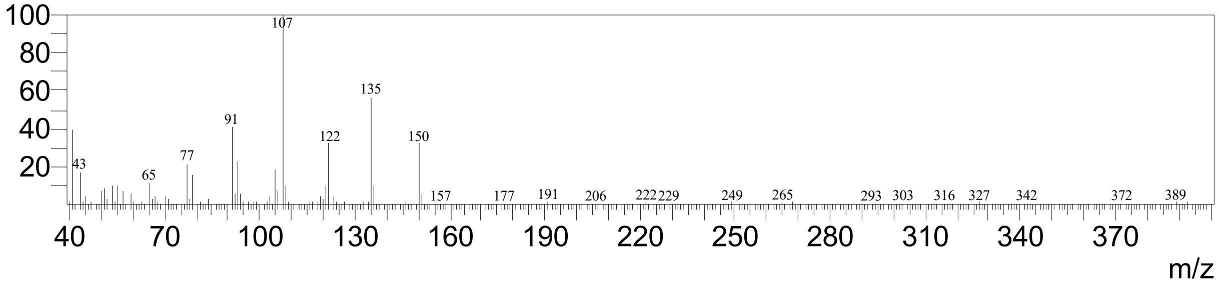

Compound: 1-Methylcamphene

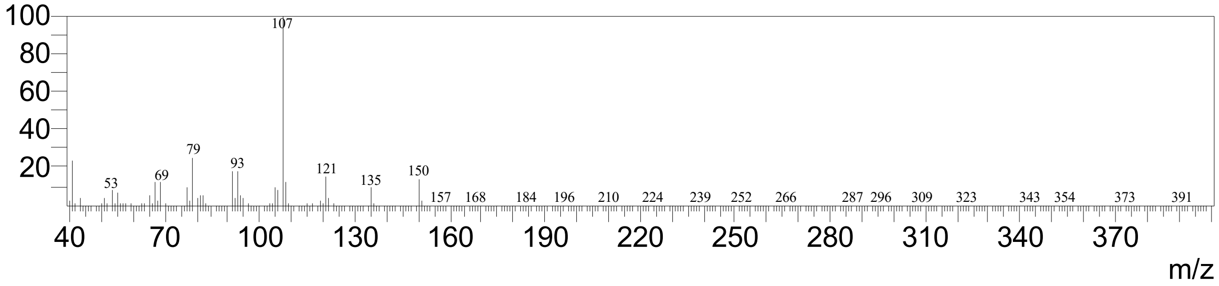

Compound: 2-Methylenebornane

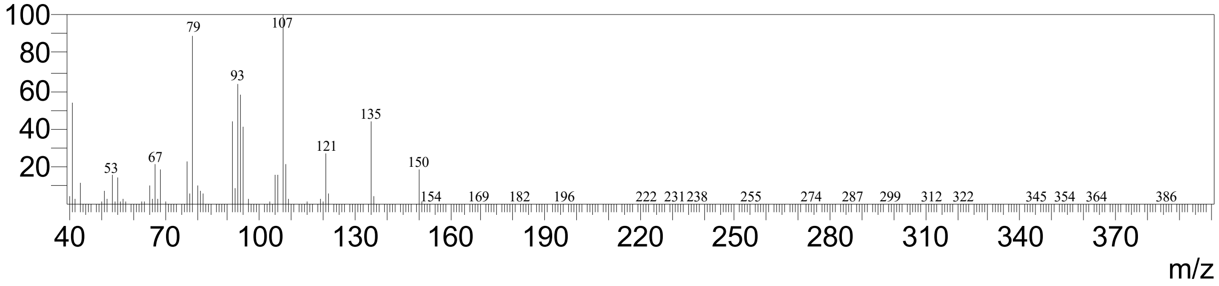

Compound: 2-Methylmyrcene

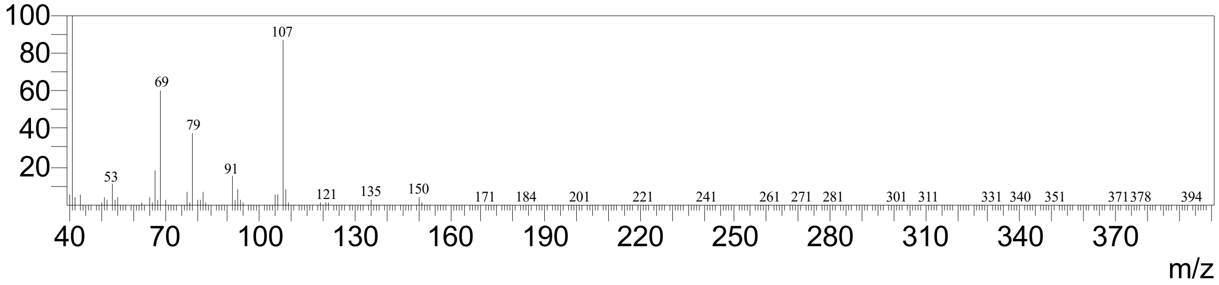

Compound: 2-Methyllimonene

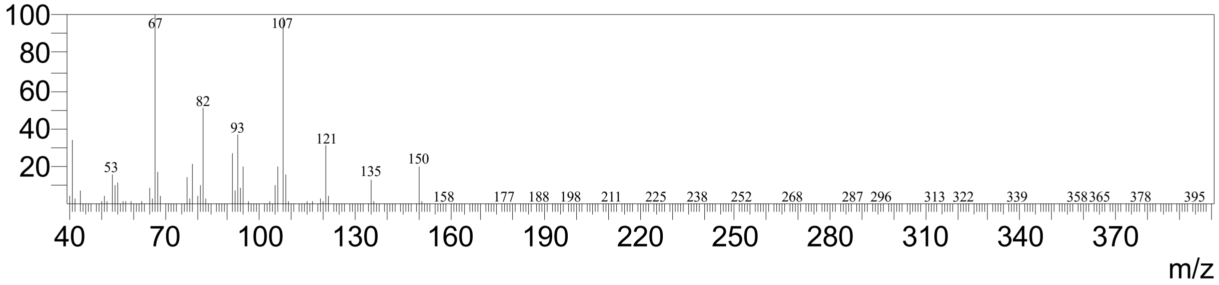

Compound: 3,4-Dimethylcumene

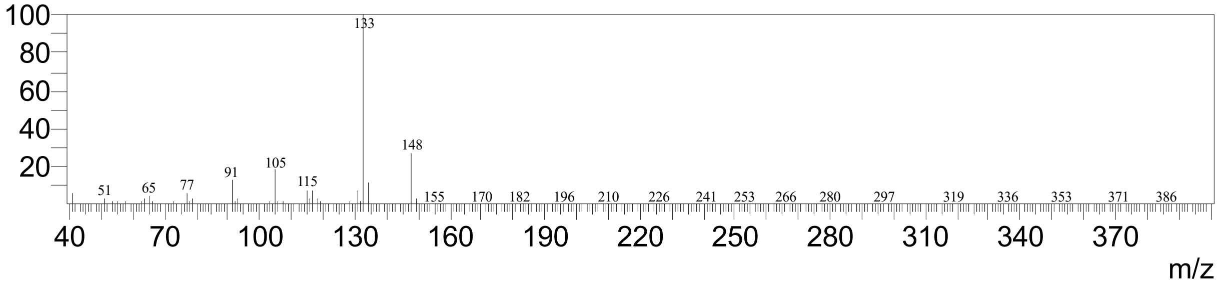

Compound: 2-Methylisoborneol

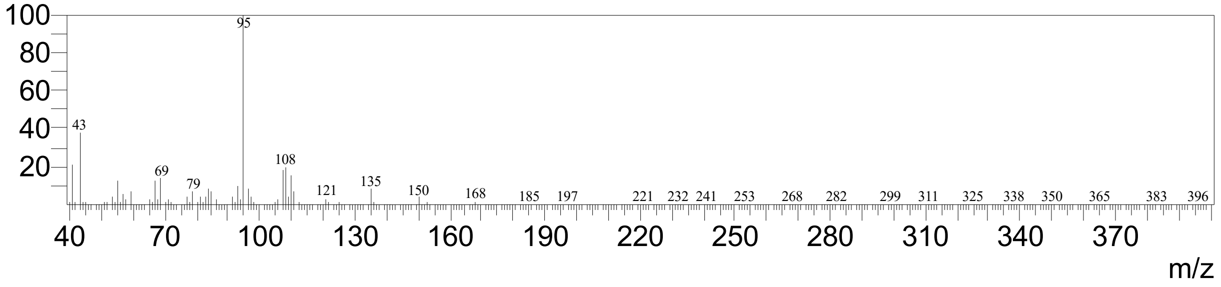

Compound: 2-Methyl-beta-fenchol

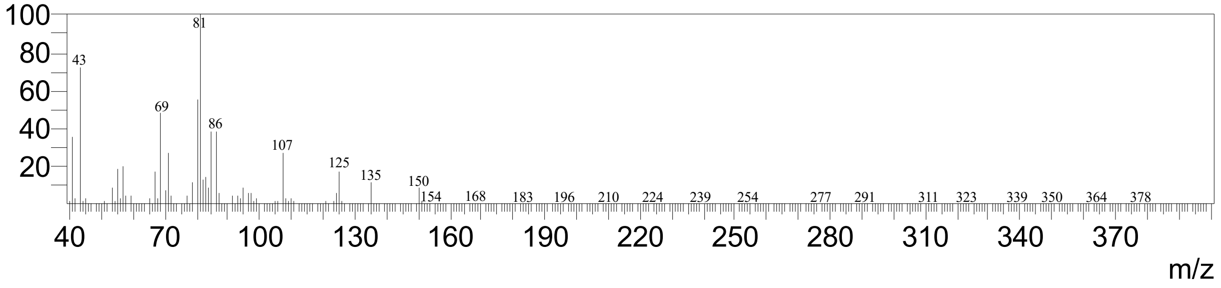

Compound: 2-Methyllinalool

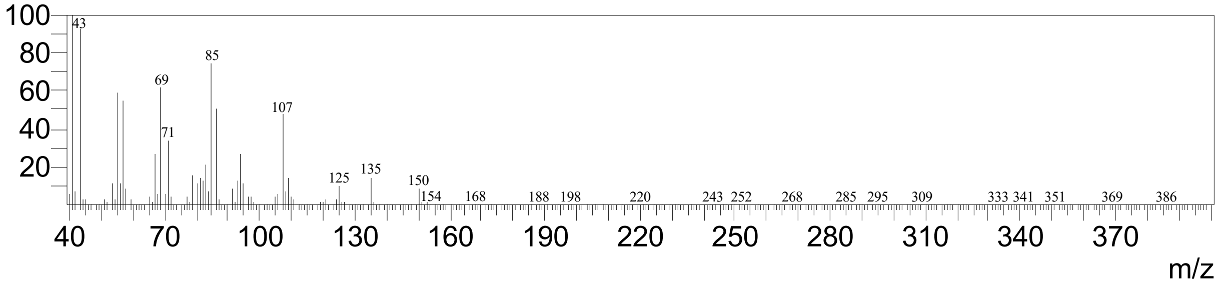

Compound: 2-Methylborneol

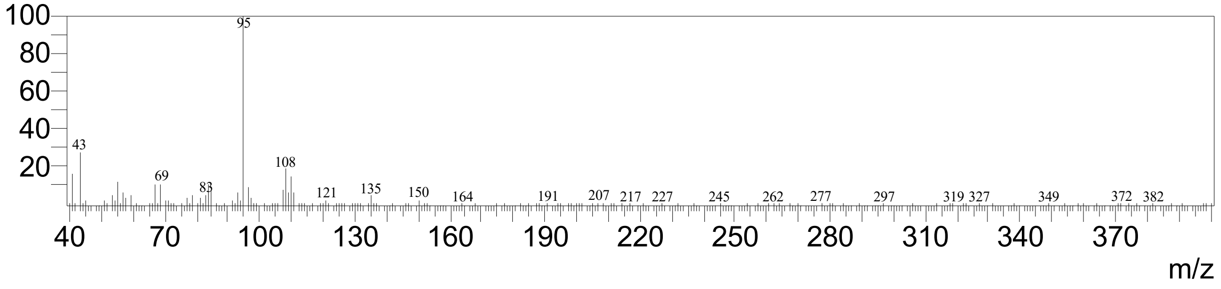

Compound: 2-Methyl-alpha-terpineol

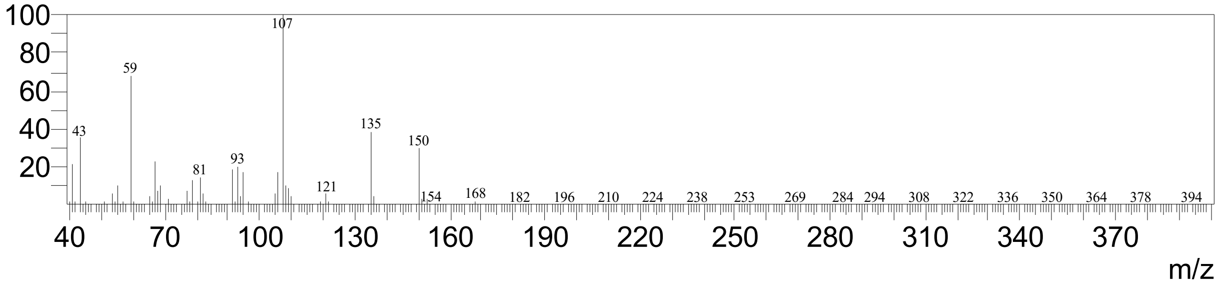

Compound: 2-Methylcitronellol diastereomer 1

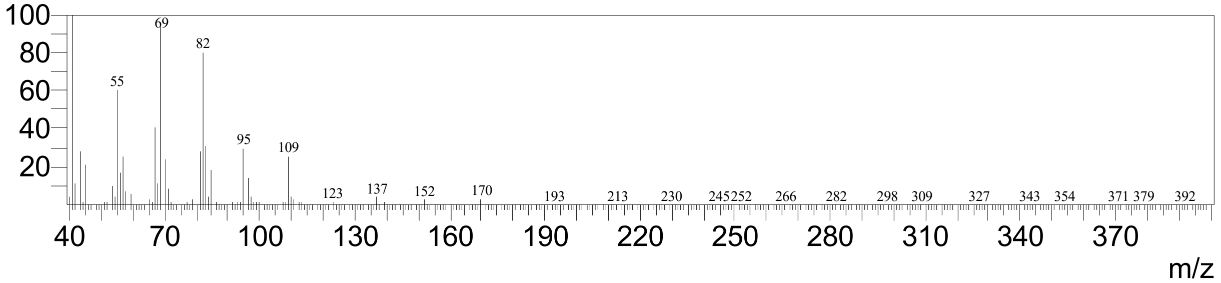

Compound: 2-Methylcitronellol diastereomer 2

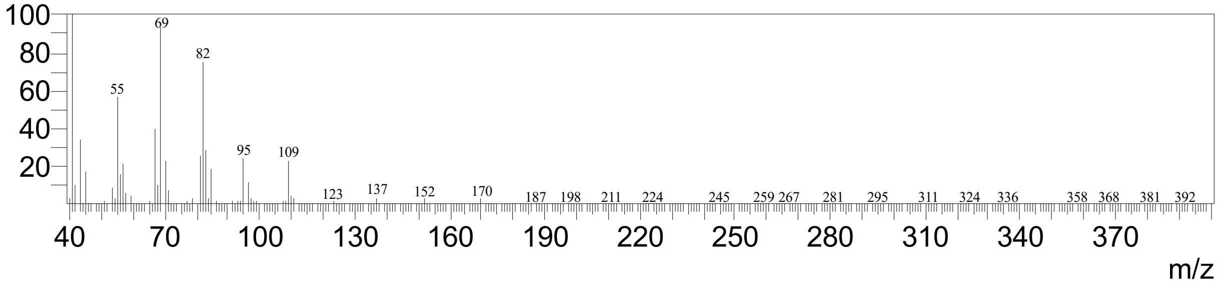

Compound: 2-Methylnerol

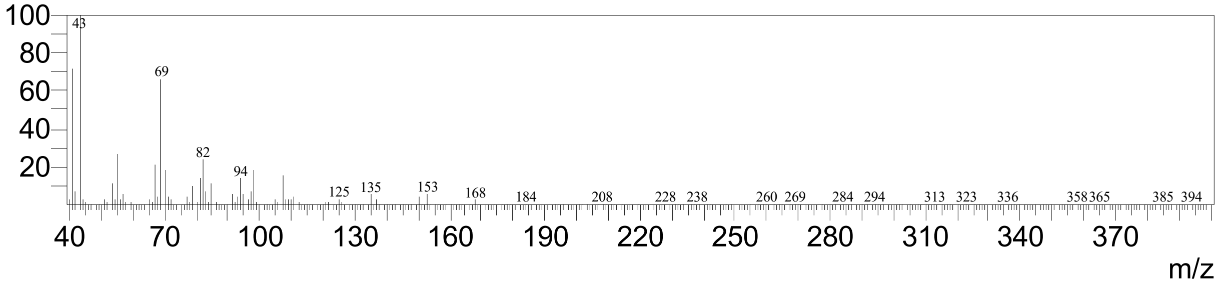

Compound: 2-Methylgeraniol

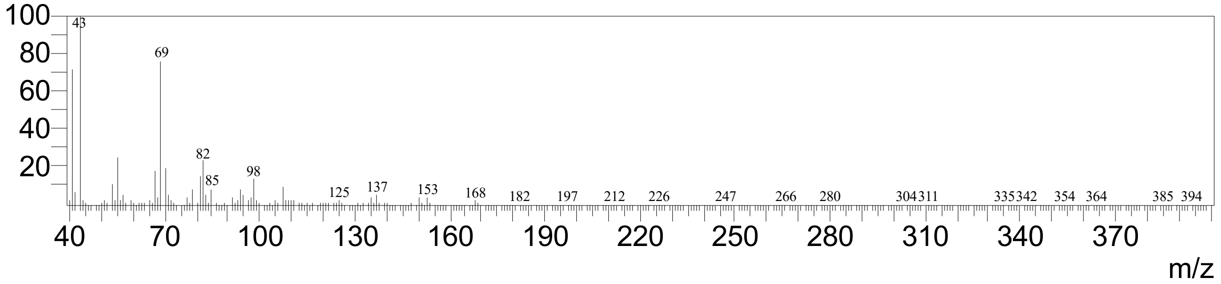

Compound: 4-Methyl-3-carene

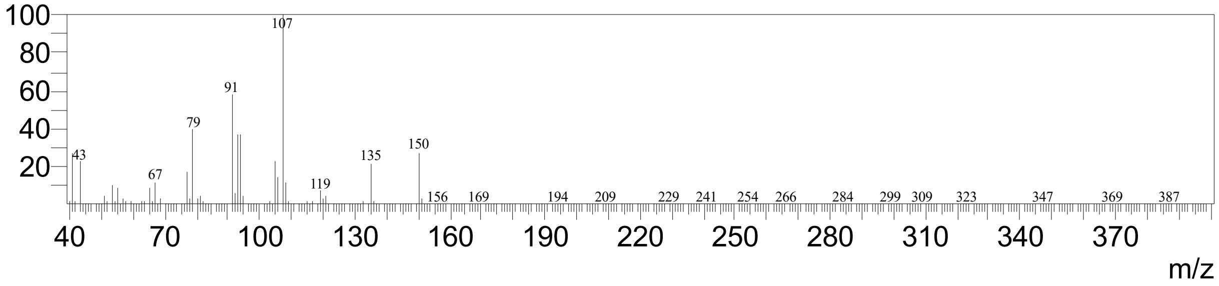

Compound: 6-Methylfarnesol

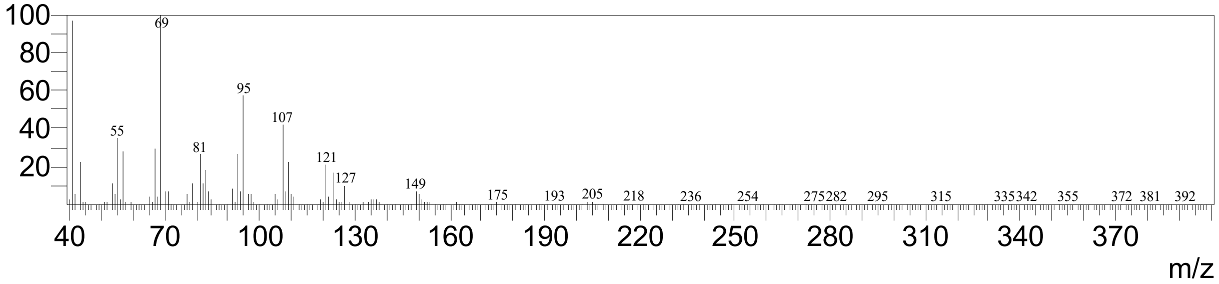

Supplement: S7 Fig — (PDF) [file pone.0196082.s009.pdf]
