# Supplementary material for: Heterologous expression of 2-methylisoborneol / 2 methylenebornane biosynthesis genes in Escherichia coli yields novel C11-terpenes
Source: PLoS One. 2018 Apr 19;13(4):e0196082. doi: 10.1371/journal.pone.0196082 (PMC5908152; doi:10.1371/journal.pone.0196082)

S8 Fig. Total ion chromatograms of HS-SPME-GCMS analyses of the control strains 0-0, 10-0 and 11-0 compared to the one of the production strain 11-p.

Labelled peaks are listed in Table 3 and S2 Table

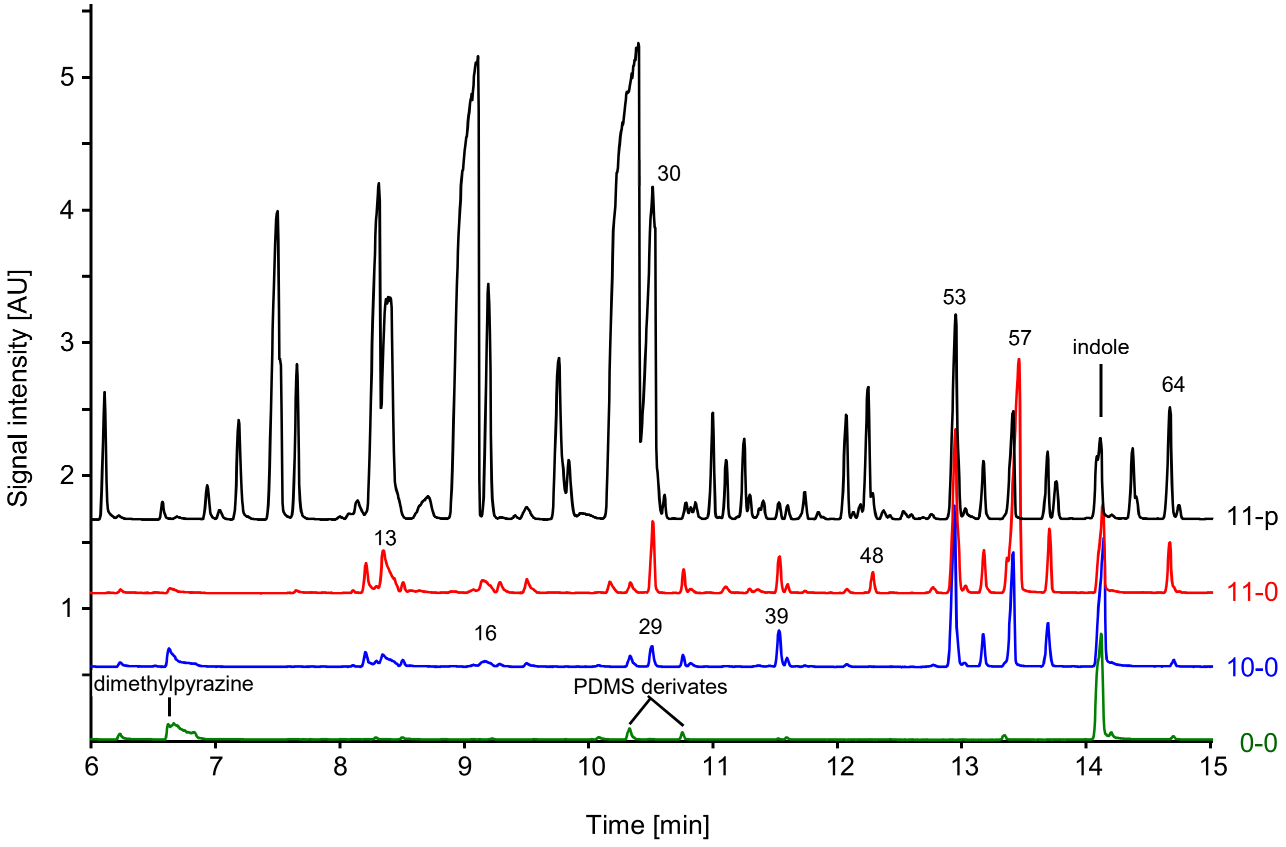

Supplement: S8 Fig — (PDF) [file pone.0196082.s010.pdf]
